# Role of Hybrid Brain Imaging in Neuropsychiatric Disorders

**DOI:** 10.3390/diagnostics5040577

**Published:** 2015-12-04

**Authors:** Amer M. Burhan, Nicole M. Marlatt, Lena Palaniyappan, Udunna C. Anazodo, Frank S. Prato

**Affiliations:** 1 St. Joseph’s Health Care London, Parkwood Institute, 550 Wellington Road, London, ON N6C 0A7, Canada; E-Mail: nicole.marlatt@sjhc.london.on.ca; 2 Department of Psychiatry, Schulich School of Medicine & Dentistry, University of Western Ontario, London, ON N6C 2R6, Canada; E-Mail: Lena.Palaniyappan@lhsc.on.ca; 3 Lawson Health Research Institute, London, ON N6C 2R5, Canada; E-Mails: uanazodo@lawsonimaging.ca (U.C.A.); prato@lawsonimaging.ca (F.S.P.)

**Keywords:** neuropsychiatric disorders, neuroimaging, humans, brain disorders/diagnosis, hybrid imaging, molecular imaging, neuronal networks, Alzheimer’s disease, schizophrenia, affective disorders

## Abstract

This is a focused review of imaging literature to scope the utility of hybrid brain imaging in neuropsychiatric disorders. The review focuses on brain imaging modalities that utilize hybrid (fusion) techniques to characterize abnormal brain molecular signals in combination with structural and functional changes that have been observed in neuropsychiatric disorders. An overview of clinical hybrid brain imaging technologies for human use is followed by a selective review of the literature that conceptualizes the use of these technologies in understanding basic mechanisms of major neuropsychiatric disorders and their therapeutics. Neuronal network abnormalities are highlighted throughout this review to scope the utility of hybrid imaging as a potential biomarker for each disorder.

## 1. Introduction

Neuropsychiatric disorders represent a group of syndromes exhibiting abnormalities in cognition, emotions and/or behaviors. The burden of neuropsychiatric disorders is substantial. The 2004 World Health Organization (WHO) report on the global burden of disease indicates a high prevalence of these disorders worldwide. These diseases affect millions of people each year: unipolar depression 151.2 million; bipolar disorder 29.5 million; schizophrenia 26.3 million; Alzheimer’s disease and other dementia 24.2 million. In all regions of the world, neuropsychiatric disorders account for about one-third of adult disability, as measured by years of healthy life lost due to living in less than optimum health [1].

### 1.1. Issues with Clinical Classification

In general, neuropsychiatric disorders tend to cluster into cognitive, affective, and/or psychotic domains with significant overlap in symptomatology across these domains. The field of psychiatry classifies mental health disorders categorically based on phenomenological criteria (symptoms and course of illness). The current classification system is based on expert consensus and listed in the *International Classification of Disease, 10th edition* (ICD-10) [2] and the fifth edition of the *Diagnostic and Statistical Manual of Mental Disorders* (DSM-5) [3]. The classification system is necessary for clinicians and researchers to communicate about these disorders, but because of the many sub-components of neuropsychiatric disorders, it has arguably fallen short in depicting the true nature of these illnesses [4]. A clinical phenomenon in neuropsychiatry can result from a variety of illnesses; for example deficits in executive function can be part of schizophrenia, but also mood disorders, attention deficit hyperactivity disorder (ADHD), or dementia. Misdiagnosis or late diagnosis of psychiatric disorders is a major challenge for clinicians. For example, in one community sample of bipolar disorder patients, approximately 70% had a missed diagnosis [5], which can have detrimental consequences for a disorder that is often associated with suicidal ideation.

The heterogeneity in presentation of neuropsychiatric disorders makes it difficult to identify specific biomarkers, which has led some experts to relate biomarkers to symptom clusters rather then to the illness as a whole [6]. Taken together, these findings highlight major dilemmas for psychiatrists trying to provide effective treatment for neuropsychiatric disorders: (1) Correctly diagnosing the underlying illness causing the symptoms; (2) Initiating and individualizing treatment as early as possible; and (3) Monitoring the impact of different therapeutics in a valid and reliable way.

### 1.2. Brain Imaging as the Biomarker for Neuropsychiatric Disorders

**Figure 1:**
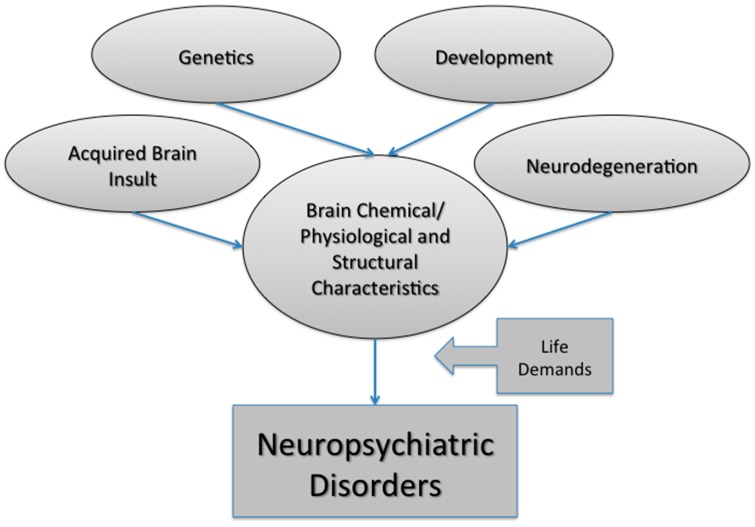
Multiple factors contribute to neuropsychiatric disorder susceptibility. These factors include genetic predisposition, developmental insult, brain injury and natural (or disease-state) aging. Together these factors can influence the molecular, physiological and structural characteristics of the brain. Environmental (or external) factors, such as the demands of life, loss of a loved one or experiencing abuse can also have a negative impact on brain health. Taken together, neuropsychiatric disorder development is extremely cofounded with multiple variables, resulting in individualized trajectories to the same end point: a neuropsychiatric disorder.

Neuropsychiatric disorders likely represent a complex interplay between biological, psychological and social factors. The final common pathway to psychiatric symptoms is usually an interaction between vulnerability and psycho-social factors, with disease vulnerability coming from several sources such as genetic/epigenetic factors, developmental insult (biological and/or psychological), acquired brain insult (traumatic, vascular, toxic, infectious *etc.*), or neurodegeneration (Figure 1).

Despite decades of research, the biological underpinnings of most neuropsychiatric disorders remain elusive. This is mainly due to the low specificity of several biomarkers investigated, rendering them less useful in identifying disorders, predicting disease progression or treatment responses. A biomarker is defined as “a characteristic that is objectively measured and evaluated as an indicator of normal biological processes, pathogenic processes, or pharmacologic responses to a therapeutic intervention” [7]. For some disorders with neuropsychiatric aspects like Huntington’s disease, genetic biomarkers can predict the diagnosis with nearly perfect certainty [8]. Genetic confirmation is expanding in other neuropsychiatric illnesses such as frontotemporal lobar degeneration (FTLD) [9] and in Alzheimer’s disease (AD) [10,11], although with significant variability in heritability patterns, pathological and clinical phenotype expression. Despite our current understanding of some mental illnesses like schizophrenia as being highly heritable [12], no reliable genetic biomarker with a clear link to disease mechanism has been identified [13].

With the brain being the organ of the mind, brain imaging received significant attention as a possible surrogate biomarker for neuropsychiatric disorders. For a neuroimaging biomarker to serve as a surrogate endpoint for the clinical phenomenon, it needs to be validated against a gold standard where there is a high level of certainty in treatment response, the projected outcome of a disease and especially in diagnosis [14]. This became a possibility in the field of AD where antemortem diagnostic markers can be verified against reliable postmortem neuropathological markers like amyloid plaques and neurofibrillary tangles [15].

The wide clinical availability of brain imaging modalities such as computed tomography (CT) and later magnetic resonance (MR) imaging resulted in a tremendous amount of literature on brain structure abnormalities in neuropsychiatric disorders. However, a recent meta-analysis of structural imaging studies by Goodkind *et al.* (2015) identified gray matter loss in dorsal anterior cingulate and the insula that was common across six distinct diagnostic groups (schizophrenia, bipolar disorder, depression, addiction, obsessive-compulsive disorder, and anxiety), with only few specific findings that distinguished depression and schizophrenia from other diagnoses [16]. This highlights the need for a better understanding of the underlying molecular changes that lead to volume loss and differential therapeutic options and highlights the difficulty in identifying specific biomarkers for each disorder by using only structural imaging acquisitions.

### 1.3. Evolution of Network Models in Neuropsychiatric Disorders

Numerous theoretical and empirical studies now approach the function of the brain from a structural and functional network architecture perspective [17]. It offers a powerful framework, which can be explored in investigating cognition and affective dysfunction in neuropsychiatric disorders. A brain network is currently defined as a set of brain systems (cytoarchitectonically or functionally distinct regions with connections between regions) that serve a specific or set of specific behaviors. The brain networks have nodes (regions or vertices) and connections between them called edges [17]. The Human Connectome project that is currently underway [18] provides great potential for learning about disease states by mapping large numbers of healthy participants in an attempt to understand the range of variability that is acceptable. It is now clear that defects in functional integration and aberrant connectivity is present in neuropsychiatric disorders [19].

Most of the brain’s cognitive functions are based on the coordinated interactions of large numbers of neurons that are distributed within and across different specialized brain areas. A fundamental, yet unresolved, problem of modern neuroscience is how this coordination is achieved. Integration and segregation of neural activity needs to occur at various spatial and temporal scales, and these scales must be dynamically adjusted depending on the nature of the respective cognitive tasks.

Brain networks on their own and as a group must work in harmony in order to regulate certain functions, including thinking (cognition), emotion, motivation, and behavior. The last few decades of brain research has been driven by advances in imaging, allowing scientists to characterize an increasing number of brain networks in hopes of better understanding human behavior. This has resulted in significant progress in identifying patterns of brain structure and function in healthy individuals and those with psychiatric disorders. Animal models of neuropsychiatric disorders offered significant advancements to understand molecular signaling in the brain across different neuronal networks. On the other hand, some highly evolved brain networks could not be modeled in available animal models. The evolution of functional neuroimaging including functional MR imaging (fMRI), Positron Emission Tomography (PET) and Diffusion Tensor Imaging (DTI) gave rise to the field of research into large-scale brain networks in neuropsychiatry [20]. Resting-state fMRI studies have repeatedly demonstrated the existence of a high degree of temporal correlations in the BOLD (blood-oxygen-level dependent) contrast imaging fluctuations of several brain regions, strengthening the notion that distinct large-scale networks constitute the physiological basis for mental functions [21,22]. While different methods of anatomical parcellation and image processing have yielded a number of variations in the make-up of these large-scale networks [23], three specific networks appear to be more consistent than the others [19,22,24]. These major core functional networks are thought to be involved in the interface between cognition and emotions and are called the default mode network (DMN), the salience network (SN) and the central executive network (CEN). The DMN is composed of central structures including the medial prefrontal cortex (mPFC) and the posterior cingulate cortex (PCC). The DMN is busy when you are mentally passive or not engaged in a specific task. The CEN includes the dorsolateral prefrontal cortex (DLPFC) and inferior parietal cortex (IPC) and is involved in attention and executive function. Unlike the DMN, the CEN is engaged in higher-order cognitive and attentional control. Lastly, the salience network (SN) is composed of the anterior insula (AI), mainly in the right side, and anterior cingulate cortex (ACC) and is involved in coordinating switching between DMN and CEN based on the individual’s needs [25].

It is thought that there is a dynamic relationship between these networks whereby the SN coordinate activation-deactivation of CEN and DMN based on emotional salience. During various brain states (*i.e.*, resting and task-performing states), the DMN and CEN respectively, show more BOLD activity than the other [21,26]. Pivotal work carried out by Sridharan *et al.* [27], implied that the anterior insula, an important hub in the SN, acts as a driver node. The preceding states of anterior insula predicted the activity in both the DMN and CEN, during both resting and task-performance states. Thus, a third brain state, the state of switching between the two other states, is associated with the activity of the SN [24].

## 2. Hybrid Imaging

Hybrid (multimodal or fusion) imaging is an integrated technology that combines functional/molecular imaging and structure imaging technologies [28]. The strengths of each modality synergistically complement each other to create a new and more powerful tool, overcoming their stand-alone limitations. Many hybrid imaging platforms are capable of true simultaneous data acquisition and therefore leads to an increased level of confidence with which disease abnormalities can be localized [29]. The potential of hybrid imaging to reveal molecular processes *in vivo*, while simultaneously depicting their anatomic location, provides many benefits to many different disciplines. Specifically, the generalized benefits include increased diagnostic accuracy, reduced radiation exposure, advancements to individualized medicine (or molecularly targeted medicine), and enables the precise monitoring of interventional procedures. These benefits are useful in oncology, cardiology, neurology, psychiatry and pharmacology for facilitating diagnosis, staging the disease, defining treatment plans, and monitoring treatment response; outcomes that anatomical imaging techniques (*i.e.*, MR imaging or CT) alone cannot provide. Furthermore, functional and metabolic changes can and do occur without a corresponding anatomical abnormality [30]. Hence, hybrid imaging will play a major role in advancing our understanding of diseases and theranostics for years to come.

There are many hybrid imaging modalities currently available: PET/CT; Single-photon emission computed tomography (SPECT)/CT; MR/PET; MR/SPECT; ultrasound/MR; ultrasound/CT; MR/CT and two different ways in which imaging modalities are combined [31]. The *software fusion* approach aligns two image sets post hoc after being acquired on different scanners at different times (reviewed by [32]). In addition, the *hardware fusion* method combines instrumentation for two imaging modalities and acquires both images within the same reference frame. This is more novel than the software approach and groundbreaking in medical imaging by being able to acquire co-registered structural and functional information of the system in a single scan. There has been a significant amount of focus on *hardware* *fusion* hybrid imaging modalities ever since tomographic imaging of function/metabolism (PET) was combined with anatomical localization (CT) and voted “Medical Invention of the Year” in 2000 by Time magazine (*Time* 4 December 2000).

### 2.1. Hybrid PET/CT: Development and Utility in Neuroimaging

Prior to the commercial introduction of hybrid PET/CT in 2001, individual PET scanners were marketed primarily for research. However, the clinical acceptance of this technology was quick to occur, with oncology being the first discipline to see the potential of PET/CT and accept its clinical capability in the early 2000s [33]. The adoption of PET/CT as a clinical tool, continued to grow so rapidly that it eventually was no longer commercially viable to market stand-alone PET systems. In fact, more than 95% of all PET scanners sold in 2004 were hybrid PET/CT scanners and by 2006, practically all stand-alone PET scanners had been replaced by PET/CT scanners.

Individual disciplines have had different levels of success and challenges they have had to face in implementing PET/CT into clinical practice. In oncology, for instance, most clinical investigations need only one PET probe and the uni-spectral nature of PET is quite acceptable with CT providing anatomical context and attenuation correction for PET quantitation. However, in cardiology, PET/CT is not ideal, as PET images can take up to 30 min or more and CT takes seconds. Specifically, physiological motion associated with the heart and lungs generally gives errors in the superposition (*i.e.*, co-registration) of the cardiac PET and CT three-dimensional data [34]. In brain imaging this registration is not a problem, provided patients do not move their head during the PET data collection or between the PET and CT imaging sessions. By 2010, international acceptance for hybrid imaging of the brain was obvious, after more than 60% of all surveyed European institutions reported using PET/CT for neurology [35].

PET relies on exogenous chemical radiolabeled molecules being injected into the bloodstream that have an affinity component and a signaling component [28]. The most widely used tracer in oncology and neurology, to date, is the PET imaging glucose analog ^18^F-fluorodeoxyglucose (^18^F-FDG). In neurology, the gray matter of the brain preferentially uses glucose as a metabolic substrate and both increased and decreased metabolism is used to evaluate neurological abnormalities.

It may appear that PET is limited by the development of safe PET radiotracers for use in humans. However, novel PET radiotracers are developed continuously and now exist for many metabolic substrates, hypoxia agents, neurotransporters, and drugs [36]. The utility of these tracers can be highlighted by certain PET studies into pre-symptomatic (or pre-clinical) Alzheimer’s disease, which have been proven to detect dementia earlier than other imaging modalities or neurological tests [37,38]. Hence, PET tracers are likely to change the way in which disease processes are understood and treated. However, the major drawback to using PET/CT imaging routinely, is patient exposure to both external radiation from the CT scan and internal radiation from the injected tracer [39]. This ultimately limits the ability to do repeat PET studies using different PET tracers, especially in healthy controls, and has generated a greater interest in non-ionizing techniques such as MR imaging.

### 2.2. Hybrid PET/MR: Development and Utility in Neuroimaging

**Figure 2:**
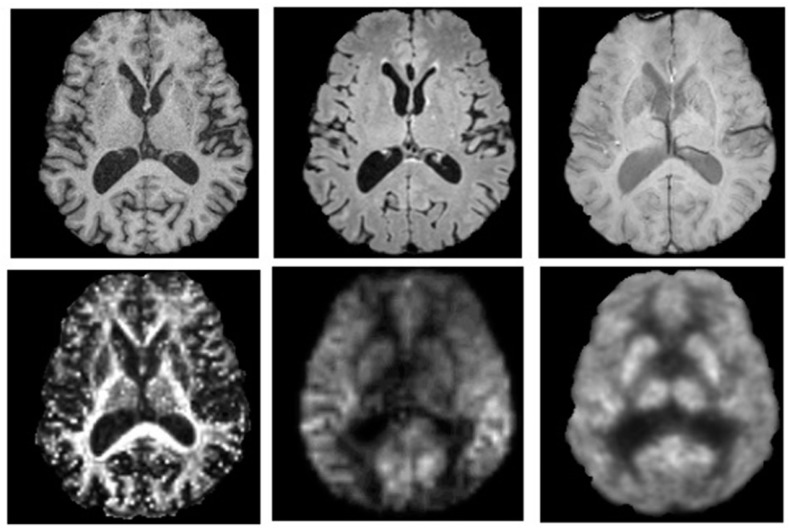
PET and MR images of a 77-year-old male injected with 198 MBq of ^18^F-flurodeoxyglucose (FDG) while patient was lying supine in a Siemens Biograph mMR PET-MR hybrid scanner (Siemens Healthcare, Erlangen, Germany). A 60 min dynamic PET scan was acquired during multispectral MR imaging. These images illustrate the capacity to perform multi-parametric mapping in a single session simultaneously, to improve characterization of neuropsychiatric conditions. Axial images are from (**top**) left to right: T1-weighted (MRPAGE) for tissue-specific volumetric measurements, T2-weighted (FLAIR) for assessment of white matter lesions, and susceptibility-weighted imaging for detection of microbleeds and cerebral amyloid angiopathy; (**bottom**) left to right: fractional anisotropy image from diffusion tensor imaging for quantification of white matter structural integrity, perfusion weighted-imaging (ASL) for hemodynamic measurements and a PET-^18^F-FDG glucose consumption image. Images are presented with permission from patient and are courtesy of Lawson imaging, Lawson Health Research Institute (LHRI), London, ON, Canada.

**Figure 3:**
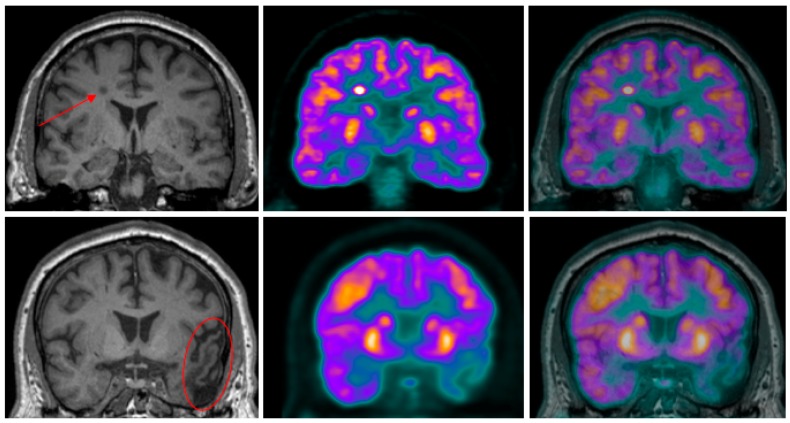
Coronal high-resolution T1-weighted MR (left) and PET (center) images of two pulmonary carcinoma patients scanned at LHRI, London, Ontario, Canada using a Siemens Biograph mMR PET-MR hybrid scanner. The PET-^18^F-FDG and MR images were acquired simultaneously and, the PET was later superimposed onto the MR images (right). To illustrate the synergistic effect of PET’s high sensitivity and MR’s high spatial resolution to improve the specificity of characterizing neuropsychiatric disorders, two case studies are presented. (**Top**) row: A focal area of increased FDG-PET uptake in a metastatic lesion can be seen on the top row images that is well defined when the PET image is overlayed onto the MR image; (**Bottom**) row: Areas of hypometabolism in PET-^18^F-FDG images, a result of prior traumatic brain injury, are delineated in the fused PET and MR images. Images are presented with permission from patients.

MR imaging is a much more complex and versatile modality, compared to CT, in terms of the different characteristics of human tissue it is able to measure. MR imaging reveals structure and function through atomic (primarily protons) interactions with a strong magnetic field. The primary method of revealing function by MR imaging is the utilization of the BOLD signal [40]. This specialized brain scan has the ability to map neural activity by imaging the hemodynamic response (blood flow) that is directly correlated to energy use by brain cells [41]. Therefore, the desire to combine this useful modality of brain mapping with PET imaging has been long-standing, though technically difficult. This difficulty arises because PET detection had been dependent, since its development, on photomultiplier tubes (PMT) that will not function in the magnetic fields associated with MR. Therefore, the major hurdle that had to be overcome was the complete redesign of the PET detector assembly such that it would be magnetically insensitive. This hurdle left PET/MR in the pre-clinical stage, until 2006 when the first simultaneous PET/MR imaging of the human brain took place [42]. Currently, many technical challenges, including possible interference between these modalities, have mostly been resolved [43] and the first commercially marketed PET/MR scanner was available by 2010. Figure 2 shows an example of simultaneous hybrid imaging scans of an older adult’s brain acquired on a commercial Siemens Biograph mMR PET/MR hybrid scanner. The synergistic nature of combining PET and MR imaging modalities is depicted in Figure 3 by showing separate PET and MR imaging scans and contrasting these with a hybrid image overlay.

MR imaging offers better contrast among soft tissues as well as functional-imaging capabilities, compared to CT. For example, PET/MR data acquisition is simultaneous, *versus* sequentially collected in PET/CT imaging. This gives PET/MR essentially perfect temporal correlation of dynamically acquired data sets from both modalities [44]. The excellent soft-tissue contrast and the fact that it reduces the effective radiation dose are additional advantages of MR for pre-clinical research studies and in clinical applications [39,43]. The major advantage of PET/MR imaging over PET/CT in neuroscience research is likely to be forthcoming within the next few years. Specifically, multi-parametric analysis of complex functions in neural networks is possible through PET/MR imaging [45]. Therefore, neurotransmitter release, combined with simultaneous functional measures (*i.e.*, changes in the BOLD signal measured by fMRI) has the potential to reveal system-level process abnormalities in the brain [46]. This is not made possible with PET/CT, as steady-state conditions are required because data is acquired sequentially *versus* simultaneously. This exciting perspective on measuring network level processes by PET/MR technology is key to understanding neuropsychiatric disorders, as research findings suggest that major psychopathologies involve dysfunction of cognitive and emotion regulation processes and effect distributed brain regions within multiple spanning lobes [19]. An integrated model of network-based cognitive and affective dysfunction in psychopathology is discussed next to frame the importance of imaging brain networks in understanding major neuropsychiatric disorders.

### 2.3. Utility of Hybrid Imaging in Understanding Brain Connectivity in Neuropsychiatric Disorders

In recent times, functional neuroimaging methods such as Magnetoencephalography (MEG) have been employed to examine the physiological basis of the salience network model [47]. Nevertheless, this mechanistic model is largely built on cross-sectional studies of BOLD signal variations in different patient groups in comparison with healthy controls. Despite the growing number of studies examining connectivity metrics using various mathematical derivatives, the physiological basis of the correlations in fluctuating BOLD signal is still unclear [48,49]. BOLD signal from a voxel in the fMRI reflects a measure of haemodynamic variations within the voxel space. Around 10^6^ neuronal cell bodies are present in a typical voxel unit observed in fMRI studies, alongside numerous glial cells that exceed neuronal count by several orders [50]. While BOLD signal fluctuations at rest are often interpreted as proxy signals for neuronal oxygen consumption (and thus neuronal activity), this has not been conclusively proven as of yet [51]. Regional variations in vascular supply, and/or neurovascular coupling are strong suspects in generating the apparent patterns of connectivity as read by BOLD signal correlations [52]. Pathological states that affect certain specialized brain regions more than the others could result in alteration of neurovascular coupling, thus resulting in patterns of perturbed connectivity. Combined PET/MR imaging that allows simultaneous study of BOLD-based connectivity and quantitative assessment of cerebral blood flow and oxygen extraction, can provide the much needed insight into the basis of dysconnectivity in the so called connectopathies such as schizophrenia and autism. In addition the role of microglial activity in dysconnectivity can be discerned by using one of the several PET ligands that focus on the study of microglia, in PET/MR hybrid imaging studies (for example, [53]).

## 3. Alzheimer’s Disease as a Prototypical Neuropsychiatric Disorder

Alzheimer’s disease (AD) represents a major public health issue with high prevalence that increases with age [54]. With reliable neuropathological biomarkers including amyloid plagues and neurofibrillary tangles and prominent cognitive and behavioral symptomatology, AD can be considered a prototypical neuropsychiatric disorder. The availability of reliable biomarkers that brain imaging could be verified against have brought brain imaging closer to diagnostic utility.

### 3.1. Large Investments Result in Tangible Progress for Neuropsychiatric Disorders

Large investment projects, such as the Alzheimer’s disease Neuroimaging Initiative (ADNI) have resulted in tangible progress in identifying reliable biomarkers. In its second phase now, the ADNI was funded with 67 million US dollars by government agencies and industry, enrolled 800 participants with mild cognitive impairment (MCI), those with early AD and healthy subjects. With over 200 publications to date, this initiative has resulted in large collections of clinical data, brain imaging information, genetic profiles and cerebrospinal fluid (CSF) sets, which have all contributed to uncovering unique disease markers. Clinical profiles can now be correlated with imaging, genetic and biochemical data, given the standardized simultaneous acquisition. Blood, CSF and molecular imaging data facilitated better profiling of the core pathology tied to the disease mechanism(s); mainly the amyloid and *TAU* (tubulin associated unit) cascades [55]. An extension of this initiative started in 2010 and will continue until 2016, with a plan to enroll an additional 550 participants. Key achievements from this initiative were summarized in 2005 and 2013 [56,57]. Briefly, a standardized protocol to acquire clinical, imaging, blood and CSF markers across multiple sites resulted in better assessment of the diagnostic utility of these markers and the added value of combining different modalities to allow testing models of the disease.

There are several published reviews and consensus papers on the utility of brain imaging in AD and related disorders. Some of the neuroimaging techniques have been recommended for clinical use such as structural image (CT or MR imaging) to rule out space occupying lesions or silent cerebrovascular lesions and to add to diagnostic certainty by examining brain atrophy patterns. Furthermore, hypoperfusion patterns and/or glucose hypometabolism can be helpful to differentiate between AD and other forms of dementia like frontotemporal dementia (FTD) (for review see [58,59]). Efforts to modify core biomarkers in AD have begun [60]. For example, trials to remove amyloid from the brain or prevent its aggregation have been conducted with anti-amyloid monoclonal antibodies. Although initial results of trials in mild to moderate AD has been clinically disappointing, there is clear evidence of removal of amyloid from the brain especially in those carrying Apolipoprotien-E4 alleles (a known susceptibility gene marker for the risk to develop AD) [61]. More trials are now underway targeting earlier stages of AD pathology, including those in the pre-clinical stage, those with genetic risk, and those showing positive PET amyloid scans [62,63]. In particular, one notable method by which the Amyloid-β mechanism is commonly explored is by employing carbon 11-labeled Pittsburgh compound B (^11^C-PiB) with PET imaging. This specific PET radiotracer can specifically bind to deposition in the human brain and is able to provide quantitative information on burden *in vivo* [64,65]. Therefore, molecular imaging is now at the forefront in the development of therapeutics targeting the underlying disease processes in AD.

### 3.2. Neuroimaging the Neuropsychiatric Characteristics of Alzheimer’s Disease

Less is known when it comes to neuroimaging correlates of neuropsychiatric symptoms of AD. In the first population-based study of a community, 75% of patients with AD and other dementia manifested at least one neuropsychiatric symptom, leading the authors to conclude that neuropsychiatric symptoms occur in the majority of persons with dementia [66]. The rate of neuropsychiatric symptoms in residential facilities, such as nursing homes, can be as high as 78% [67]. Neuropsychiatric symptoms of dementia include several domains and can be clinically rated with the Neuropsychiatric Inventory (NPI) scale [68]. These symptoms can be clustered under affective (apathy, depression, anxiety, euphoria, irritability, disinhibition), psychotic (delusions, hallucinations) or behavioral (agitation, aberrant motor function, eating and sleep abnormalities). These symptoms are a core part of AD and result in significant distress to patients, their caregivers and the system of care. They can also result in grave consequences including rapid deterioration, earlier institutionalization, and faster mortality [69]. Attempts to treat these symptoms with psychotropic medications that are used to treat primary psychiatric illnesses yield mixed and at best modest efficacy, but with significant increased risk for strokes and mortality [70].

#### 3.2.1. Psychosis and Alzheimer’s Disease

Only recently have basic mechanism of neuropsychiatric symptoms of AD been studied with neuroimaging. Psychosis studies of AD including delusions and hallucinations have reported volume loss in gray matter of brain areas involved in large-scale brain networks (CEN, DMN and SN). When it comes to delusions in AD, gray matter volume loss has been reported in the AI [71,72] and ACC [73], both components of the SN. Evidence also suggest the involvement of structures in the CEN in delusions of AD including left and right inferior frontal areas, left middle frontal area, left inferior parietal lobule and left claustrum [74] and lateral frontal and parietal areas [73]. In a systematic review including 25 studies using imaging to investigate delusions in AD, Ismail *et al.* (2012) concluded that the majority of studies implicated right-sided predominance involving mainly frontal areas in paranoid delusions, but involving temporal areas in misidentification delusions that are thought to be different in origin [75]. Only few studies examined change in metabolic signal in the brain on those with AD and delusions. In this regard Sultzer *et al.* (2014) reported hypometabolism (^18^F-FDG-PET study) in the right lateral frontal cortex, orbitofrontal cortex, and bilateral temporal cortex in patients with delusions and AD. They also reported that low cortical metabolic activity in bilateral medial frontal cortex was associated with poor insight [76].

When it comes to hallucinations in AD, evidence extracted from the ADNI database identified relative gray matter volume loss in the anterior part of the right insula, left superior frontal gyrus and lingual gyri and relative hypometabolism in a large right ventral and dorsolateral prefrontal area in AD patients with hallucinations compared to those without. The “core region” identified as being associated with hallucinations was the right anterior part of the insula, which is the central node in SN. Four of their AD-hallucination group had mixed AD and alpha-synclein pathology prominent in Lewy Body Dementia, which demonstrates the need for a better understanding of the underlying molecular changes in the affected areas and related network [77]. Others reported that reduced supramarginal cortical thickness was predictive of increasing hallucinations over time, further highlighting the issue with specificity of structural imaging findings [78].

#### 3.2.2. Agitation and Alzheimer’s Disease

Other neuropsychiatric symptoms on AD that received some attention in neuroimaging literature include agitation. This symptom can be difficult to define, given that it can arise for different reasons and can be driven by unmet physical or mental needs [79,80]. Areas representing the SN are affected in “agitation” of AD. These include the ACC and AI [74,81]. Using connectivity as a biomarker, Balthazar *et al.* (2014) reported hyperconnectivity between ACC and right insula in patients with AD and agitation [82]. A summary of imaging findings by Rosenberg *et al.* (2015) speculated that agitation in AD is associated with deficits in structure and function of the frontal cortex, ACC, posterior cingulate cortex, amygdala, and hippocampus and may be associated with mechanisms underlying misinterpretation of threats and affective regulation [83].

#### 3.2.3. Apathy and Alzheimer’s Disease

Apathy is a common symptom in AD. It involves the lack of concern and motivation, diminished emotionality and goal directed activities from a lack of motivation, but without emotional distress [84,85]. This symptom has been associated with gray matter atrophy in many areas of the brain, including the left and right ACC [74]. Reduced baseline inferior temporal cortical thickness has also been found to be predictive of increasing apathy over time in a mixed cohort of 812 community dwelling individuals including healthy controls, MCI and AD [78]. MCI patients with apathy had significantly decreased metabolism in the posterior cingulate cortex in a study using ADNI database ^18^F-FDG-PET data [86]. A study by Lanctot *et al.* (2007) compared non-depressed patients with AD and apathy verses those with AD and no apathy and found that apathy was associated with hypoperfusion in the left ACC and right orbitofrontal cortex with relative hyperperfusion of the right and left hippocampi, left medial superior temporal gyrus, and the middle medial temporal cortex [87]. Taken together the three large-scale brain networks are also involved in apathy, but again the specificity of this involvement needs to be clarified.

### 3.3. Network Neuroimaging Limitations in Alzheimer’s Disease

While molecular imaging of amyloid, tau cascades and other targets has made significant progress in therapeutic trials by targeting the underlying pathology of AD, there have been no such studies reported on imaging neuropathological correlates of neuropsychiatric symptoms of AD. Postmortem pathological studies have shown that AD patients with psychosis tend to have higher phosphorylated tau in frontal areas [88] and co-occurrence of alpha-synclein pathology [89]. However, no imaging studies have looked at these *in vivo* as potential biomarkers of neuropsychiatric symptoms of AD as of yet.

There are several limitations in the neuroimaging literature around neuropsychiatry of AD. In addition to limitations inherent in the clinical definition of these symptoms, the majority of studies used volumetric measures as a main outcome. These measures use cross-sectional identification methods to assess relative loss of gray matter in patients with and without neuropsychiatric symptoms. This does not take into account the severity of the underlying neurodegeneration and other co-morbidities such as vascular factors and mixed neuropathology (for example alpha-synclein). Areas identified as having volume loss and part of the neurodegenerative process are responsible for a wide range of cognitive and psychiatric symptoms, making it difficult to sort out the contribution of these changes to neuropsychiatric symptoms specifically. Areas responsible for these symptoms have been identified in the CEN, DMN and SN, but despite the evidence available from perfusion scans and metabolism scans, we do not have information about the molecular changes involved in structural and functional changes.

There is emerging evidence for abnormalities in the SN in other forms of dementia such as fronto-temporal dementia (FTD). This illness is characterized by a dramatic decline in interpersonal function including lack of empathy, disinhibition, apathy, executive dysfunction, aberrant motor function and compulsive fixations with relative preservation of memory encoding function [90,91]. With the early behavioral changes, volume loss in areas involved in the SN has been reported (right AI, pregenual anterior cingula, and amygdala) [92]. More work is needed to investigate neuroimaging correlates in other forms of dementia including Lewy Body disease, which has hallucinations as a core feature [93] in order to understand the underlying mechanism of neuropsychiatric symptom profiles in dementia-related illnesses.

## 4. Psychoses

Psychotic disorders (or psychoses) are major mental disorders that cause abnormal thoughts and perceptions in the sufferer. Schizophrenia is a type of psychotic disorder, but other illnesses such as bipolar disorder (BD) or AD can also be accompanied with psychotic symptoms. The two main symptoms of psychosis are delusions (false beliefs) and hallucinations (false perceptions), such as feeling, seeing or hearing something that is not there. For several decades now, dopaminergic manipulation has been the primary source of therapeutic symptom relief in patients experiencing hallucinations and delusions. Despite the immediate relief in psychotic symptoms brought on by dopaminergic blockade, in clinical practice several patients do not show adequate response to these agents. In addition, a substantial number of patients who initial show a positive response to these medications tend to relapse, despite the continued use of antipsychotic agents [94].

### 4.1. Network Abnormalities in Psychosis

A number of multimodal imaging studies now consistently point towards an abnormality in the function of the SN in various psychiatric disorders [19], especially psychosis [95]. The lack of a smoothly operating function switching mechanism among various task-specific networks and between executive and DMN could provide a neural model for disease, explaining the plethora of seemingly unrelated psychiatric symptoms that occur together in syndromes such as schizophrenia [96,97,98,99]. Experimental studies point towards an association between cortical and subcortical dysconnectivity and the severity of psychotic symptoms [100]. In patients, the degree of failure in the ability of the SN to influence other networks has been shown to predict the severity of psychosis that persists despite the use of antipsychotics [98]. This raises the possibility that non-dopaminergic circuits may have a crucial role in the phenomena of treatment resistance, persistence and vulnerability to relapse, and the failure of coordinated activity among crucial brain networks. This may provide the biological substrate underpinning that influences poor outcomes in psychosis. Currently, many PET radiotracers specific to the evaluation of the dopamine system, as well as the involvement of other monoamines (glutamate, gamma-aminobutyric acid (GABA)) in the human brain are currently available [101,102,103]. Hybrid imaging opens up the possibility of examining both dopaminergic and non-dopaminergic contributions to dysfunctional large-scale networks in psychosis.

### 4.2. Multimodal Imaging Opportunities in understanding Molecular and Functional Attributes of Psychosis

Schizophrenia is increasingly viewed as a long-term illness that progresses through critical clinical stages in most if not all patients [104]. Circumstantial evidence from MR spectroscopy (MRS) studies indicates that in early stages of schizophrenia, a glutamatergic excess may be seen, which later evolves into a state of glutamatergic deficit [105]. In line with this observation, stage specific changes in connectivity are also noted in schizophrenia. In particular, a failure to deactivate the DMN appears to develop during early stages of the disease [106], with more widespread and pronounced deficits in chronic and established illness [107]. Combined PET/MR spectroscopy imaging across various illness stages in schizophrenia can throw more light into the association between changes in glutamatergic levels and the patterns of large-scale network level connectivity in the brain.

The inhibition/excitation balance between GABA and glutamate system has been hypothesized to be abnormal in schizophrenia [108]. Numerous postmortem studies point to a widespread deficiency in the GABA interneuron distribution [109]. These post-mortem observations, in addition to a growing body of GABA imaging studies in psychosis [110,111] firmly point to a perturbation in GABA levels in schizophrenia. A missing link in this line of argument is how the distributed abnormalities in GABA function relate to the myriad of symptoms seen in psychosis. Computational approaches that mathematically model inhibition and excitation in combination with fMRI observations have provided some clues as to the relationship between network-level dysconnectivity and synaptic-level transmitter abnormalities seen in psychosis [112]. Recent studies using serial PET and fMRI imaging suggest that GABA receptor activity is crucial in inducing the negative BOLD response seen in DMN regions in the presence of external task-processing demands in healthy subjects [113], but these observations are limited by the lack of simultaneous readings from GABA-PET and BOLD signals. Hybrid PET/MR imaging offers the potential to further investigate the network-level mechanism through which GABA dysfunction influences the symptoms of psychosis. In addition to being an important pathophysiological enquiry in psychosis, these studies can also offer surrogate endpoints to study the pharmacological efficiency of putative GABA or glutamate modulating agents in psychosis.

In recent times, there is a renewed interest in the role of brain inflammation involving microglial response in the pathophysiology of schizophrenia [114]. Two important lines of evidence add strength to these arguments:

(1) NMDA (*N*-Methyl-d-aspartate) encephalitis, an autoimmune inflammatory condition, mimics schizophrenia in many subjects and has been identified as an important, albeit small, contributor of first episode psychosis in clinical practice [115].
(2) Several NSAID’s (nonsteroidal anti-inflammatory drugs) have shown to have a small but noticeable effect in reducing symptoms of schizophrenia [116].

PET imaging offers the ability to study microglial activation; combined with simultaneous fMRI imaging, this can be a powerful tool to narrow the distance between putative inflammatory response in the brain and the distinct cognitive disturbances that are typical of neuropsychiatric disorders such as schizophrenia. Many opportunities for *in vivo* molecular imaging of neuroinflammation are currently available through the development of PET radiotracers and have recently been reviewed by Ory *et al.* (2014) [117].

## 5. Affective Disorders (Mood and Anxiety Disorders)

Affective Disorders are a set of psychiatric illnesses that are also referred to as mood disorders. The main types of affective disorders are anxiety disorders, unipolar depression, and bipolar disorder. Symptoms vary by individual, but they typically affect mood. The symptom-based diagnostic approach used for affective disorders is an obstacle in diagnosis, as anxiety disorders have tremendous overlap and are commonly comorbid with depression [118,119]. Further, a single manic episode changes the diagnosis from unipolar depression to bipolar disorder, which is thought to have a specific pathophysiological underpinning.

From a molecular neurobiology approach, affective disorders seem to pose a unique challenge. Serotonin is the neurotransmitter that is best studied and is responsible for regulating mood, anger, reward, aggression and appetite [120]. Serotonergic neurotransmission is altered in neuropsychiatric disorders such as depression, anxiety, bipolar disorders, autism, schizophrenia and AD [121]. However, the cause of depression and other affective disorders is far from being a simple deficiency in central monoamines. For example, the immediate increase in monoamine transmission from selective serotonin reuptake inhibitors (SSRIs) or monoamine oxidase inhibitors (MAOIs) does cause a mood enhancing property (over time), but it is clear from additional studies on healthy controls that a direct reduction in monoamines does not directly alter mood [122]. Therefore, there are currently many hypotheses regarding the pathophysiology of affective disorders at the molecular level and further research is needed in this area to develop a mechanistic framework in which therapeutic targets and early diagnostics can be enriched. Advances in research applications of neuroimaging technology may have a future in clinical applications of imaging biomarkers for establishing diagnosis and predicting illness course or treatment outcomes for affective disorders [14].

### 5.1. Anxiety Disorders

There are six major types of anxiety disorders: generalized anxiety disorder (GAD), obsessive-compulsive disorder (OCD), panic disorder (anxiety attacks) (PD), specific phobia (SP), post-traumatic stress disorder (PTSD), and social anxiety disorder (SAD). Similar to other neuropsychiatric disorders, anxiety disorders develop from a complex set of risk factors, including genetics, brain chemistry, personality, and life events. Anxiety disorders are also one of the most prevalent categories of psychopathology [123] and share symptoms of irrational fear, abnormal behavior, hyperarousal, excessive anxiety and avoidance to triggers. These disorders are plagued by comorbidities that impede treatment attempts [124] and cause problems in correct diagnosis. Recent advancements in neuroimaging may help distinguish certain anxiety disorders and serve as a potential diagnostic and therapeutic biomarker for this group of disorders. PTSD and OCD are no longer included in the anxiety disorder category in the new DSM-5 system [125], but because of the shared features (excessive fear, avoidance and hyperarousal) and comorbid nature with other anxiety disorders, they will be included here as a category of anxiety disorder.

### 5.2. Anxiety Disorders and Network Abnormalities

Functional connectivity research into anxiety disorders is very sparse. However, a recent review on anxiety disorders by Peterson *et al.* (2014) attempts to conceptualize whether resting-state connectivity could provide diagnostic specificity, and reveal neurobiological distinctions between the anxiety disorders [126]. Similar to schizophrenia and AD research, there are recent studies that suggest that anxiety disorders can be characterized by functional network connectivity abnormalities that exist within and between the large-scale brain networks (DMN, SN and CEN). In PTSD, for example, abnormalities within and between the DMN, and the SN appear to be predominant. In particular, PTSD has been shown to be associated with reduced functional connectivity within the DMN (reviewed by Peterson *et al.* (2014)) [126] and increased connectivity within the SN (between insula and other SN regions including the amygdala) [127]. PTSD participants also demonstrated increased inter-network connectivity, or abnormally reduced segregation between the DMN and SN [128]. These functional connectivity studies explain the disrupted attention and increased threat sensitivity in PTSD individuals. The increased coupling within the SN, at rest, reduced coupling within the DMN and the increased cross-network connectivity between the large networks suggests that salience detection *versus* internally focused thoughts are not at equilibrium in PTSD clients and may explain the hyperarousal symptoms evident in PTSD sufferers during task-free conditions.

In social anxiety disorders (SAD), the most consistent research findings to date suggest that the CEN functional connectivity is decreased within this network [129]. There is also preliminary evidence that SAD is associated with decreased connectivity between the CEN and the DMN [129]. Additional resting-state functional connectivity studies are necessary for further understanding the large inter-network connectivity that are associated with SAD and provide an explanation as to why others have reported mixed findings regarding the large network connection patterns [126]. This will be especially important for understanding the resting-state intra- and inter-connectivity of the amygdala (a subcortical structure of the SN), as it has been strongly associated with an individual’s response to fear [130,131].

In OCD, the DMN has been found to have decreased intra-connectivity [132,133] suggesting that OCD patients have dysfunctions of self-referential mental activities [134,135]. Thus, disrupted DMN intra-connectivity is likely to be involved in the psychopathological symptoms of OCD. There is also evidence that OCD is associated with an increased intra-connectivity within the SN [136]. These findings are similar to that found with PTSD. This is consistent with the conclusion drawn from Peterson *et al.* (2014): that there appears to be a certain degree of overlap within the neural networks underlying different anxiety disorders [126]. Future studies into functional brain connectivity should involve comparisons between different anxiety disorders, such that specificity between the disorders can be established.

The richest information in decoding the molecular and functional specificity in anxiety disorders could come from PET/MR hybrid imaging studies of the DMN. Considering that the default mode network is thought to engage in introspection, which involves moving away from externally focused thoughts while initiating internal, self-focused thoughts; the diverging DMN connectivity profiles of affective disorders will uncover differences in introspective processes. DMN hyperconnectivity in affective disorders is thought to relate to excessive introspection and thus internal focus in the form of rumination [137], whereas externally focused thoughts related to OCD or PTSD requires greater attention to potentially threatening externally focused thoughts [138]. To further investigate these aspects, DMN connectivity profiles, coupled with specific PET radiotracers (*i.e.*, dopamine, glutamate, noradrenaline, and GABA) should be directly compared between affective disorders in future studies. These investigations may explain treatment resistance prevalence of affective disorders [139,140] and comorbidity correlates that are prevalent in these illnesses [141,142].

### 5.3. Depression

Depression is a complex neuropsychiatric disorder with diverse aetiologies and generally the onset is idiopathic. It is also multifactorial (heterogeneous, genetic associations, environmentally influenced, and affects multiple regions of the brain) with no definitive neurobiological correlates. The core symptoms of depression include depressed mood, diminished interests, appetite changes, low concentration, sleep dysregulation, psychomotor changes, loss of energy, feeling of worthlessness and excessive guilt, diminished concentration, and recurrent thoughts of suicide [3]. Currently, depression is diagnosed exclusively from behavioral observations where 5 of the 9 symptoms must be present. Therefore, clinical judgment is crucial in diagnosing depression, such that substance abuse and physiological effects of medical conditions are considered. However, the symptom-based diagnostic approach is an obstacle in diagnosis, as anxiety overlaps as a common comorbidity with depression [118,119] and a single manic episode changes the depression diagnosis to bipolar disorder, which is thought to have a specific pathophysiological underpinning.

### 5.4. Predictive Biomarkers for Depression

Despite almost sixty years of neurobiological research into depression, there remains to be no definitive blood/CSF or neuroimaging finding that can serve as a reliable biomarker. Genetic associations have not uncovered strong and consistent “depression genes”, likely because of its heterogeneity. However, many candidate genetic predispositions are known, though their overlap with other disorders and influence from the environment to initiate depression makes them unlikely candidates for clinical diagnosis [143,144]. The array of risk factors for depression has forced researchers to adopt a heterogeneous disease concept for depression pathophysiology. The polysyndromic nature of depression and heterogeneous nature of this condition demands the need for multiple-technical approaches to explore the neurobiological bases for depression. Combining imaging techniques, such as hybrid imaging, will be able to capture the “big picture” of the disease rather than offering only a single pixel at a time; a common result from single modality techniques. Nonetheless, the abundance of research that involves single neuroimaging techniques, capturing one molecule at a time or by recording increases and decreases of regional brain activity is noteworthy for depression. The individual pieces of information revealed through previous imaging techniques could offer a conceptual framework for neurobiological correlates to be uncovered, especially if these findings parallel the current understanding of depression as a network-based disorder [145].

The neurobiological cause(s) for depression are currently not definitively known and has resulted in a lack of reliable diagnostic, or therapeutic biomarkers [146]. However, research suggests certain molecules such as monoamine serotonin, norepinephrine, dopamine, glutamate and GABA maybe the most promising. Neuropeptides such as neuropeptide Y, neurokinin/substrate P and galanin have also been associated with the pathology of the more severe and debilitating form of depression called major depressive disorder (MDD) [147].

### 5.5. Network Abnormalities in Depression

Altered activity in several distributed brain networks may help explain hypervigilance, ineffective emotional regulation, maladaptive rumination and poor executive control associated with MDD [148]. Researchers have therefore become interested in the role large-scale functional network communication plays in the pathophysiology of MDD [149,150]. The functional networks have correlated brain activity at rest and during tasks. Cognitive and emotional processes most affected in depression are correlated with the frontoparietal network (FPN), the DMN, and the SN [22]. Dysfunction in the DMN can cause disruption to internal attention and the SN can cause emotional processing and the monitoring of salience events to be dysregulated, which may correlate best with the cognitive and affective functioning deficits seen in depression.

A recent meta-analysis reinforces the hypothesis that depression is a network-based disorder [151]. The study provides the first cohesive evidence that MDD is associated with abnormal connectivity within and between brain networks that are associated with internal/external attention, and emotional (salience) functioning. The analysis consistently found: (1) Increased connectivity within the anterior DMN; (2) Increased connectivity between the SN and the anterior DMN; (3) Changed connectivity between the anterior and posterior DMN; and (4) Decreased connectivity between the posterior DMN and the CEN. The model proposed fits well with network deficits being linked to regulating attention and mood for MDD sufferers [152]. Imbalanced network functioning may be connected to diminished cognitive control, deficits in goal-directed behavior and a preference toward internal thoughts rather than the external world [151].

The network model of depression is an active field of research with multiple advancements being consistently uncovered. For example, potential diagnostic and treatment biomarkers are emerging from functional connectivity studies. Chen *et al.* (2015), recently found a direct association between the initial onset of depression and the internal functional connectivity of the DMN [153]. This suggests that specific region-to-region connectivities may serve as a diagnostic biomarker for the initial onset of MDD. Another promising biomarker could be associated with the functional connectivity between the precuneus (a central node of the DMN) and other distant regions of the brain. For example, Peng *et al.* (2015) discovered that depression severity could be associated with functional correlations measured by fMRI when focusing on the central node of the DMN [154]. Lastly, a functional network analysis of specific regions in the DMN has shown a high level of reproducibility in predicting early therapeutic improvement of MDD patients. This may serve as a potential biomarker for guiding personalized therapeutic regimens in MDD [155]. Taken together, these recent advancements suggest depression is a network-based disorder. Future studies into the DMN of depressed subjects should incorporate hybrid imaging (PET/MR) techniques, such that underlying aberrant molecular aspects of the disorder are coupled to the functional properties of the large networks. With objective treatment-specific biomarkers on the horizon for the treatment response in depression [156], information gained by combining imaging modalities may lead to definitive biomarker development that can eventually lead to clinical applications.

### 5.6. Bipolar Disorder (BD)

Bipolar Disorder is a chronic disabling neuropsychiatric disorder that effects millions of individuals worldwide across their lifespan [1] and a significant cause of increased mortality and morbidity, making it one of the leading causes of disability worldwide [157]. The disorder is complicated by tremendous heterogeneity, and influenced by genetic and environmental factors [158]. BD can be characterized by recurrent cycles of mania and depression episodes separated by normal mood and classified into four subtypes (bipolar I disorder; bipolar II disorder; cyclothymic; and not otherwise specified) [159]. The symptomology of BD overlaps anxiety disorders, affective disturbances, cognitive deficits, and high incidence of somatic and psychiatric comorbidity [160]. Diagnosing bipolar disorder can therefore be a difficult task, even for physicians with many years of expertise [161]. The disorder often exhibits symptomology (affective instability, neurovegetative abnormalities, cognitive impairments, psychosis, and impulsivity) found in other psychiatric disorders, which often leads to misdiagnosis or late diagnosis of the disorder. As many as 80% of patients are misdiagnosed within the first year of treatment [162], which can be devastating to patients who often experience suicidal tendencies. In one community sample of diagnosed bipolar disorder patients, one third of the participants went 10 years or more without a correct diagnosis. In addition, these patients had on average 3.5 other diagnoses and saw on average four physicians before receiving the correct diagnosis [5]. Taken together, it is clear that undiagnosed BD results in significant patient suffering and a substantial economic burden.

The complexity, heterogeneity, cofounding illnesses and absence of clinical diagnostic markers for BD is a major dilemma for psychiatrists trying to provide effective treatment, in early diagnosis and in initiating early treatment. Strategic experiments that can reveal specific biomarkers associated with BD are much needed. Recent “omics” studies have revealed candidate biological markers for diagnosing BD. However, most studies conducted have compared BD subjects with only healthy controls, additional studies comparing potential BD biomarkers against other neuropsychiatric disorders that have overlapping symptomology (e.g., MDD) are much needed in determining specific biomarkers for BD [163].

### 5.7. Network Abnormalities in Bipolar Disorder

Very few studies have used resting-state fMRI techniques to look at network abnormalities in BD. The few reports that have come forth suggest that BD is associated with reduced connectivity within the DMN network [164]. Even less is known regarding the inter-network connectivities, though Magioncalda *et al.* (2015) has published findings that suggest that information transfer between the large networks of the brain are abnormal in bipolar disorder [165]. Two deficits were revealed between network connectivity of the DMN and the SN and explain abnormal shifting either towards internal thoughts or towards external stimuli. These connectivity abnormalities between the DMN and the SN may serve as predictive biomarkers for the manic and depressive phases of BD.

Bipolar disorder and schizophrenia share many genetic contributions [166] and overlapping clinical characteristics, including psychosis, depression and mania. This overlap can result in schizophrenia initially being misdiagnosed as an affective disorder [167]. Schizophrenia and mania have a number of symptoms and epidemiological characteristics in common, and both respond to dopamine blockade. Therefore, comparative studies between network connectivity in BD and schizophrenia have also been undertaken [168,169,170,171]. Onger *et al.* (2010) found that the medial prefrontal cortex, an area within the DMN, is abnormal in both schizophrenia and bipolar and suggest that the spontaneous oscillations observed in large-scale neuronal networks are abnormal in these psychiatric conditions, possibly underlying aspects of psychopathology [169]. Meda *et al.* (2012) reported both shared resting-state network connectivity in schizophrenia and psychotic bipolar disorder, as well as unique patterns of connectivity in each disorder [170]. A more generalized, whole brain connectivity analysis by Argyelan *et al.* (2014) has revealed novel evidence that schizophrenia and BD are whole brain connectivity disorders [172]. Specifically, when comparing global connectivity among those with schizophrenia, BD and health controls, this group found patients with schizophrenia had significantly lower global connectivity compared with healthy controls, whereas patients with bipolar disorder had intermediate global connectivity that was significantly different from those with schizophrenia and healthy controls [172]. These findings support the hypothesis that schizophrenia and bipolar disorder represent a continuum of global dysconnectivity in the brain. Hybrid imaging that incorporates neuronal activity (PET) and functional connectivity (fMRI) may facilitate diagnostic biomarker development and increase confidence in distinguishing schizophrenia from BD.

## 6. Discussion

Neuropsychiatric disorders represent a complex class of disorders that are dependent on many variables, including biological and social factors (Figure 1). They create a unique challenge for psychiatrists because they are commonly comorbid with each other and have overlapping symptomology. Clinicians must rely on their expertise and clinical judgment in order to reach a diagnosis. With large initiatives such as the ADNI we have learned that specific biomarkers for diagnosis are possible for complex disorders that encompass neuropsychiatric symptomology. Clinical profiles for MCI and AD are now possible by standardizing protocols that involve subject data collection from multiple modalities [57]. For other neuropsychiatric disorders such as depression and schizophrenia, a multimodality approach also needs to be developed. This may involve collecting clinical symptomology, obtaining blood/CSF samples, performing genomics tests and utilizing novel hybrid imaging techniques that provide details on brain structure, function and molecular processes.

### 6.1. Hybrid PET/MR Opportunities

Imaging has had a small role in diagnostic evaluation of neuropsychiatric diseases in the past. Specifically, it has been used to rule-out structural brain abnormalities, such as atrophy, neoplasm, hematoma and other surgically treatable conditions that can cause psychiatric symptomatology. However, it is now clear that hybrid imaging may provide a substantial amount of information to the molecular, structural and functional aspects of the brain. In a recent study with subjects that had neurodegenerative disease, Schwenzer *et al.* (2012) was able to simultaneously perform PET/MR imaging [173]. The results of this study showed that the MR image quality was diagnostic in all cases for neurodegenerative diseases. The researchers also state with confidence that the simultaneous imaging technique is possible with generally good imaging quality and that the imaging method allows for molecular, anatomical and functional data collection with uncompromised MR image quality and a high accordance of PET results between PET/MR and PET/CT. In moving forward, the major advancement that could come from neuroimaging would be in the identification of specific biomarkers for each particular neuropsychiatric disorder, which also serves in differential diagnosis. This advancement is on the horizon with novel hybrid imaging modalities, but neuroimaging is currently not standard clinical practice for psychiatric disorders [14].

Advances in science have been driven by both ideas and new instrumentation [174]. When PET and MR were brought together a lot was known about each modality and many results could be anticipated (driven by ideas). What is needed when considering neuropsychiatric disorders is an approach that as a first principle builds into the design that PET/MR investigations are simultaneous not sequentially acquired and that MR, as the multi-spectral component, will be used for imaging that was in the past solely a PET domain. This includes the obvious such as tissue perfusion (blood flow) with MR imaging (e.g., arterial spin labelling (ASL) [175]) rather than radiolabelled water (^15^O-H_2_O), but also for other quantities, which are found at concentrations greater than 10^−3^ M. Consider for example the benefit of measuring glucose by MR (glucose chemical exchange saturation transfer) and hence allowing PET instead to image a molecular marker of psychiatric disease such as serotonin. With such new approaches we will discover the relationship between different brain circuits and systems. Within the next decade hybrid PET/MR will help us address current unknowns such as in schizophrenia, the interaction of the dopaminergic system (by PET) with the glutamatergic system (by MR); in major depression the relationship between brain blood flow (by MR) and the serotonin system (by PET); in the differentiation of unipolar and bipolar disease; the extent of disruption of the DMN and/or the emotional encoding network (by MR) as compared to the affinity of the reward network (by dopamine receptor PET).

In the past decade there has been an explosion in the use and validation of PET tracers in dementia [176] with significant abilities to separate the different stages of dementia and understand the cause of disease. The next decade will see very significant advances to the psychiatric diseases with respect to the development of new PET tracers and understanding neuro-networks by both PET and MR imaging. However, the development of these new PET probes needs to be undertaken with the knowledge that they will be used in the context of MR. For example if it is assumed that MR quantitates brain blood flow and the brain networks, then unique information needs to come from PET. More than likely, PET imaging utility will be revealed through its higher sensitivity to molecules 10^−9^ to 10^−12^ M *versus* MR imaging (10^−4^ M) [177]. This will prove very instrumental to molecular abnormalities in neuropsychiatric disorders that mostly occur at lower concentrations (10^−8^ M). In fact, the high sensitivity of PET is currently the only *in vivo* technique available or method capable of quantifying cerebral pathophysiological subtle changes that precede neuro-structural abnormalities [178]. Therefore, the development of novel PET radiotracers will play a large role in the success of hybrid imaging.

### 6.2. Multifactorial Illnesses Require Multiple Modalities to Uncover Useful Biomarkers

In complex psychiatric disorders that have heterogeneous endophenotypes, genetic variance, and multiple brain structural/functional abnormalities, it is fair to conclude that single modality research approaches are unlikely to be able to uncover useful diagnostic, therapeutic or prognostic biomarkers for these conditions. A single genetic test works effortlessly for predicting Huntington’s disease, as it is caused by an inherited defect in a single gene [8]. However, despite tremendous efforts, uncovering specific genetic variance for neuropsychiatric disorders has not been successful. For example, despite all efforts, thus far, no single genetic variation has been identified to increase the risk of depression substantially. Genetic variants are expected to have only small effects on overall disease risk, and multiple genetic factors in conjunction with environmental factors seem to be necessary for the development of neuropsychiatric disorders such as MDD [143,144,179]. Hence, all the cofounding variables that contribute to neuropsychiatric disorders, makes the diseases multifactorial. This calls for a multimodal method of study. Hybrid imaging is a promising multimodal approach to uncovering neuropsychiatric disorder biomarkers. In 2011, Zhang *et al.* heeded this call by combining three modalities (MR/PET-^18^F-FDG and CSF) in creating a powerful biomarker for discriminating between AD and MCI [180]. By successfully utilizing multiple modalities, diagnostic biomarkers for AD and MCI exhibited remarkable sensitivity and specificity in comparison to individual modality biomarkers. Similarly, as each neuropsychiatric disorder is likely to have multiple molecular targets, radiotracer development and studies involving multiple PET targets will facilitate biomarker development in complex neuropsychiatric disorders. Progression in understanding AD, for example, was only significantly advanced when the ability to detect both beta-amyloid and various forms of the tau protein became available [181,182].

### 6.3. Therapeutic Biomarker Discovery with Hybrid Imaging

Pharmacological fMRI studies have clarified the mechanisms through which psychotropic agents such as antipsychotics and antidepressants could bring about cognitive and behavioral changes in healthy individuals and patients [183]. A major shortcoming with this approach is the lack of means to quantify the neurochemical effect caused by the pharmacological agent while measuring the changes in network connectivity or regional changes in BOLD signals. In the presence of appropriate ligands for PET, hybrid imaging can offer a powerful means to reduce the noise in pharmaco-fMRI studies and potentially uncover personalized responses to treatment. This may provide advancements in uncovering therapy monitoring biomarkers for specific psychotropic medications and shed light on their detailed mechanistic properties. For example, the question of neurodegeneration being associated with schizophrenia has been revived by some recent compelling evidence showing a progressive reduction in cortical gray matter in patients treated with continuous prescriptions of antipsychotic medications [184]. The utility of hybrid molecular imaging can be extended to biomarker discovery for therapeutic monitoring to assess an individual’s response to treatment. Specifically, molecular imaging is the only method for determining receptor occupancy [185] and drug concentrations in plasma can differ 10-fold in patients with identical administered doses. Imaging can therefore facilitate therapeutic drug monitoring, which moves towards personalized medicine and individualized dosing, reducing non-responders, and negative side-effects [186].

### 6.4. Uncovering Molecular Mechanisms with Hybrid Imaging

There are many unanswered questions when it comes to the underlying molecular aspects of neuropsychiatric disorders and the therapeutic mechanisms in which they are treated. At present it is unclear whether the reduction in cortical gray matter is an appropriate response that aids in symptom relief or an adverse consequence of the use of dopaminergic agents [187]. Several confounding factors such as the severity of illness, persistence of symptoms (despite treatment) and variations in the age of onset of the illness could mediate the observed relationship. PET/MR imaging can be used to tackle several research questions in this context. Firstly, the presence of neuronal hypometabolism (identified using PET), in regions showing greatest gray matter reduction, will support the notion that predominant loss of neuronal cells, rather than glia, accompanies the gray matter changes. Second, the role of microglial activation in this tissue loss can be studied using microglial PET markers in combination with high resolution structural MR imaging. Third, clarifying the relationship between dopamine receptor occupancy in an individual and the degree of gray matter loss can provide more direct evidence of the role of dopamine blockade in this tissue loss.

### 6.5. Hybrid Imaging as a Potential Biomarker for Neuropsychiatric Disorders

The search of biomarkers for the diagnosis and treatment of neuropsychiatric disorders has been a tedious task with low-yield of results. Genetic markers, for example, may be thought of as the best measure, as they are not altered by the disease state or treatment. In addition they are relatively easy to measure by obtaining blood sample or swabs. Nevertheless, in diseases such schizophrenia, genetic markers (especially, single nucleotide polymorphisms) have largely been disappointing, mostly due to the heterogeneity in the genetic etiological factors [188]. As a result, individual markers are of very low effect size, and not very useful in clinical practice. Some promising genetic markers (e.g., copy number variations), have larger effect sizes in case-control studies [189], but suffer from low frequency of occurrence, which renders them inadequate for diagnostics or theranostics. Markers that rely upon more proximal pathways of symptom production (e.g., large-scale brain changes studied using neuroimaging) do not suffer from the diffusion of effect size that results from etiological heterogeneity. In addition, the use of powerful multivariate statistical approaches such as pattern classification methods has increased the potential translational value of neuroimaging biomarkers. A meta-analysis of several early stage “development” studies that aim to identify the most promising predictors of diagnosis of schizophrenia, report that individual neuroimaging modalities such as fMRI or structural MR imaging have a moderate level of diagnostic performance (approximately 80% sensitivity and specificity) [190]. Importantly, the various brain changes picked-up by divergent neuroimaging tools are not mutually exclusive variables. This lack of independence means that a simple combination of the various observations may result in over-fitting and poor reproducibility of diagnostic or prognostic markers. The unknown degree of dependence among individually discriminative predictor variables makes calibration of brain-based biomarker models a challenging task. This problem calls for calibrating neuroimaging tools to enable simultaneous estimates of brain function, structure, chemical composition and metabolic activity in order to build and test valid indices that predict disease status in a given individual. Hybrid imaging provides an opportunity and optimism towards this goal.

## 7. Conclusions

Each psychiatric disorder described here should be viewed as a complex puzzle with multiple pieces; where individual pieces to the puzzle are uncovered through different techniques or modalities. Novel hybrid imaging modalities can uncover molecular, functional and anatomical aspects of each disorder and therefore have the potential to uncover multiple aspects of each neuropsychiatric disorder. We respectfully acknowledge that imaging technologies may not provide all of the necessary diagnostic, therapeutic or disease progression biomarkers needed for treating all brain disorders, though its potential to uncover specificity between disorders is warranted.

We conclude by suggesting that multimodality approaches are the best way to study multivariant neuropsychiatric disorders. Hybrid imaging will serve as one of these instrumental tools. One method of uncovering uniqueness in each disorder may be by focusing on the three main large-scale brain networks (DMN, CEN and SN), as they explain the multiple symptoms of these disorders and have shown specificity for each disorder. For these illnesses that have many cofounding influences and often comorbidities with other neuropsychiatric disorders, it is safe to assume that hybrid imaging will not give us all the answers. However, in combination with other techniques like CSF collection, genetic predispositions, convergent functional genomics, clinical attributes *etc.* we are likely to discover useful biomarkers with high specificity and sensitivity for each disorder, similar to the recent developments made for Alzheimer’s disease.

## References

[1] World Health Organization (2004). Global Burden of Disease. http://www.who.int/healthinfo/global_burden_disease/GBD_report_2004update_part3.pdf.

[2] World Health Organization (2015). International Statistical Classification of Diseases and Related Health Problems.

[3] American Psychiatric Association (2013). DSM-5: Diagnostic and Statistical Manual of Mental Disorders.

[4] Hyman S.E. (2008). A glimmer of light for neuropsychiatric disorders. Nature.

[5] Hirschfeld R.M., Lewis L., Vornik L.A. (2003). Perceptions and impact of bipolar disorder: How far have we really come? Results of the national depressive and manic-depressive association 2000 survey of individuals with bipolar disorder. J. Clin. Psychiatry.

[6] Kapur S., Phillips A.G., Insel T.R. (2012). Why has it taken so long for biological psychiatry to develop clinical tests and what to do about it?. Mol. Psychiatry.

[7] Biomarkers Definitions Working Group (2001). Biomarkers and surrogate endpoints: Preferred definitions and conceptual framework. Clin. Pharmacol. Ther..

[8] MacDonald M.E., Ambrose C.M., Duyao M.P., Myers R.H., Lin C., Srinidhi L., Barnes G., Taylor S.A., James M., Groot N. (1993). A novel gene containing a trinucleotide repeat that is expanded and unstable on huntington’s disease chromosomes. Cell.

[9] Rohrer J.D., Guerreiro R., Vandrovcova J., Uphill J., Reiman D., Beck J., Isaacs A.M., Authier A., Ferrari R., Fox N.C. (2009). The heritability and genetics of frontotemporal lobar degeneration. Neurology.

[10] Serretti A., Olgiati P., de Ronchi D. (2007). Genetics of Alzheimer’s disease. A rapidly evolving field. J. Alzheimer’s Dis..

[11] Brindle N., George-Hyslop P.S. (2000). The genetics of Alzheimer’s disease. Methods Mol. Med..

[12] Sullivan P.F., Kendler K.S., Neale M.C. (2003). Schizophrenia as a complex trait: Evidence from a meta-analysis of twin studies. Arch. Gen. Psychiatry.

[13] Bondy B. (2011). Genetics in psychiatry: Are the promises met?. World J. Biol. Psychiatry.

[14] Savitz J.B., Rauch S.L., Drevets W.C. (2013). Clinical application of brain imaging for the diagnosis of mood disorders: The current state of play. Mol. Psychiatry.

[15] Jack C.R. (2012). Alzheimer disease: New concepts on its neurobiology and the clinical role imaging will play. Radiology.

[16] Goodkind M., Eickhoff S.B., Oathes D.J., Jiang Y., Chang A., Jones-Hagata L.B., Ortega B.N., Zaiko Y.V., Roach E.L., Korgaonkar M.S. (2015). Identification of a common neurobiological substrate for mental illness. JAMA Psychiatry.

[17] Sporns O. (2012). From simple graphs to the connectome: Networks in neuroimaging. NeuroImage.

[18] Van Essen D.C., Ugurbil K., Auerbach E., Barch D., Behrens T.E., Bucholz R., Chang A., Chen L., Corbetta M., Curtiss S.W. (2012). The human connectome project: A data acquisition perspective. NeuroImage.

[19] Menon V. (2011). Large-scale brain networks and psychopathology: A unifying triple network model. Trends Cogn. Sci..

[20] Williamson P.C., Allman J.M. (2012). A framework for interpreting functional networks in schizophrenia. Front. Hum. Neurosci..

[21] Raichle M.E., MacLeod A.M., Snyder A.Z., Powers W.J., Gusnard D.A., Shulman G.L. (2001). A default mode of brain function. Proc. Natl. Acad. Sci. USA.

[22] Seeley W.W., Menon V., Schatzberg A.F., Keller J., Glover G.H., Kenna H., Reiss A.L., Greicius M.D. (2007). Dissociable intrinsic connectivity networks for salience processing and executive control. J. Neurosci..

[23] Thomas Yeo B.T., Krienen F.M., Sepulcre J., Sabuncu M.R., Lashkari D., Hollinshead M., Roffman J.L., Smoller J.W., Zollei L., Polimeni J.R. (2011). The organization of the human cerebral cortex estimated by intrinsic functional connectivity. J. Neurophysiol..

[24] Menon V., Uddin L.Q. (2010). Saliency, switching, attention and control: A network model of insula function. Brain Struct. Funct..

[25] Fox M.D., Zhang D., Snyder A.Z., Raichle M.E. (2009). The global signal and observed anticorrelated resting state brain networks. J. Neurophysiol..

[26] Fox M.D., Snyder A.Z., Vincent J.L., Corbetta M., van Essen D.C., Raichle M.E. (2005). The human brain is intrinsically organized into dynamic, anticorrelated functional networks. Proc. Natl. Acad. Sci. USA.

[27] Sridharan D., Levitin D.J., Menon V. (2008). A critical role for the right fronto-insular cortex in switching between central-executive and default-mode networks. Proc. Natl. Acad. Sci. USA.

[28] Hricak H., Choi B.I., Scott A.M., Sugimura K., Muellner A., von Schulthess G.K., Reiser M.F., Graham M.M., Dunnick N.R., Larson S.M. (2010). Global trends in hybrid imaging. Radiology.

[29] Hicks R., Lau E., Binns D. (2007). Hybrid imaging is the future of molecular imaging. Biomed. Imaging Interv. J..

[30] Anazodo U.C., Shoemaker J.K., Suskin N., Ssali T., Wang D.J., St Lawrence K.S. (2015). Impaired Cerebrovascular function in coronary artery disease patients and recovery following cardiac rehabilitation. Front. Aging Neurosci..

[31] Townsend D.W. (2008). Multimodality imaging of structure and function. Phys. Med. Biol..

[32] Slomka P.J. (2004). Software approach to merging molecular with anatomic information. J. Nucl. Med..

[33] Beyer T., Townsend D.W., Brun T., Kinahan P.E., Charron M., Roddy R., Jerin J., Young J., Byars L., Nutt R. (2000). A combined PET/CT scanner for clinical oncology. J. Nucl. Med..

[34] Cook R.A., Carnes G., Lee T.Y., Wells R.G. (2007). Respiration-averaged CT for attenuation correction in canine cardiac PET/CT. J. Nucl. Med..

[35] Cuocolo A., Breatnach E. (2010). Multimodality imaging in Europe: A survey by the European association of nuclear medicine (EANM) and the European society of radiology (ESR). Eur. J. Nucl. Med. Mol. Imaging.

[36] Zimmer L., Luxen A. (2012). PET radiotracers for molecular imaging in the brain: Past, present and future. NeuroImage.

[37] Bourgeat P., Chetelat G., Villemagne V.L., Fripp J., Raniga P., Pike K., Acosta O., Szoeke C., Ourselin S., Ames D. (2010). Beta-amyloid burden in the temporal neocortex is related to hippocampal atrophy in elderly subjects without dementia. Neurology.

[38] Rowe C.C., Ackerman U., Browne W., Mulligan R., Pike K.L., O’Keefe G., Tochon-Danguy H., Chan G., Berlangieri S.U., Jones G. (2008). Imaging of amyloid beta in Alzheimer’s disease with 18F-BAY94-9172, a novel PET tracer: Proof of mechanism. Lancet Neurol..

[39] Brix G., Beyer T. (2005). PET/CT: Dose-escalated image fusion?. Nucl. Med..

[40] Huettel S.A., Song A.W., McCarthy G. (2009). Functional Magnetic Resonance Imaging.

[41] Logothetis N.K., Pfeuffer J. (2004). On the nature of the bold fmri contrast mechanism. Magn. Reson. Imaging.

[42] Schlemmer H.P., Pichler B.J., Schmand M., Burbar Z., Michel C., Ladebeck R., Jattke K., Townsend D., Nahmias C., Jacob P.K. (2008). Simultaneous MR/PET imaging of the human brain: Feasibility study. Radiology.

[43] Disselhorst J.A., Bezrukov I., Kolb A., Parl C., Pichler B.J. (2014). Principles of PET/MR imaging. J. Nucl. Med..

[44] Pichler B.J., Judenhofer M.S., Pfannenberg C. (2008). Multimodal imaging approaches: PET/CT and PET/MRI. Handbook of Experimental Pharmacology.

[45] Garibotto V., Heinzer S., Vulliemoz S., Guignard R., Wissmeyer M., Seeck M., Lovblad K.O., Zaidi H., Ratib O., Vargas M.I. (2013). Clinical applications of hybrid PET/MRI in neuroimaging. Clin. Nucl. Med..

[46] Catana C., Drzezga A., Heiss W.D., Rosen B.R. (2012). PET/MRI for neurologic applications. J. Nucl. Med..

[47] Brookes M.J., Hall E.L., Robson S.E., Price D., Palaniyappan L., Liddle E.B., Liddle P.F., Robinson S.E., Morris P.G. (2015). Complexity measures in magnetoencephalography: Measuring “disorder” in schizophrenia. PLoS ONE.

[48] Hutchison R.M., Hashemi N., Gati J.S., Menon R.S., Everling S. (2015). Electrophysiological signatures of spontaneous bold fluctuations in macaque prefrontal cortex. NeuroImage.

[49] Ogawa S., Menon R.S., Tank D.W., Kim S.G., Merkle H., Ellermann J.M., Ugurbil K. (1993). Functional brain mapping by blood oxygenation level-dependent contrast magnetic resonance imaging. A comparison of signal characteristics with a biophysical model. Biophys. J..

[50] Logothetis N.K. (2008). What we can do and what we cannot do with fMRI. Nature.

[51] Schulz K., Sydekum E., Krueppel R., Engelbrecht C.J., Schlegel F., Schröter A., Rudin M., Helmchen F. (2012). Simultaneous bold fMRI and fiber-optic calcium recording in rat neocortex. Nat. Methods.

[52] Kim S.-G., Ogawa S. (2012). Biophysical and physiological origins of blood oxygenation level-dependent fmri signals. J. Cereb. Blood Flow Metab..

[53] Van Berckel B.N., Bossong M.G., Boellaard R., Kloet R., Schuitemaker A., Caspers E., Luurtsema G., Windhorst A.D., Cahn W., Lammertsma A.A. (2008). Microglia activation in recent-onset schizophrenia: A quantitative (*R*)-[^11^C]PK11195 positron emission tomography study. Biol. Psychiatry.

[54] Alzheimer’s Association (2014). 2014 Alzheimer’s disease facts and figures. Alzheimer’s Dement..

[55] Hardy J. (2006). Alzheimer’s disease: The amyloid cascade hypothesis: An update and reappraisal. J. Alzheimer’s Dis..

[56] Mueller S.G., Weiner M.W., Thal L.J., Petersen R.C., Jack C., Jagust W., Trojanowski J.Q., Toga A.W., Beckett L. (2005). The Alzheimer’s disease neuroimaging initiative. Neuroimaging Clin. N. Am..

[57] Weiner M.W., Veitch D.P., Aisen P.S., Beckett L.A., Cairns N.J., Green R.C., Harvey D., Jack C.R., Jagust W., Liu E. (2013). The Alzheimer’s disease neuroimaging initiative: A review of papers published since its inception. Alzheimer’s Dement..

[58] Soucy J.P., Bartha R., Bocti C., Borrie M., Burhan A.M., Laforce R., Rosa-Neto P. (2013). Clinical applications of neuroimaging in patients with Alzheimer’s disease: A review from the fourth Canadian consensus conference on the diagnosis and treatment of dementia 2012. Alzheimer’s Res. Ther..

[59] Burhan A.M., Bartha R., Bocti C., Borrie M., Laforce R., Rosa-Neto P., Soucy J.P. (2013). Role of emerging neuroimaging modalities in patients with cognitive impairment: A review from the canadian consensus conference on the diagnosis and treatment of dementia 2012. Alzheimer’s Res. Ther..

[60] Salloway S., Mintzer J., Weiner M.F., Cummings J.L. (2008). Disease-modifying therapies in Alzheimer’s disease. Alzheimer’s Dement..

[61] Salloway S., Sperling R., Fox N.C., Blennow K., Klunk W., Raskind M., Sabbagh M., Honig L.S., Porsteinsson A.P., Ferris S. (2014). Two phase 3 trials of bapineuzumab in mild-to-moderate Alzheimer’s disease. N. Engl. J. Med..

[62] Mills S.M., Mallmann J., Santacruz A.M., Fuqua A., Carril M., Aisen P.S., Althage M.C., Belyew S., Benzinger T.L., Brooks W.S. (2013). Preclinical trials in autosomal dominant AD: Implementation of the DIAN-TU trial. Rev. Neurol..

[63] Sperling R.A., Rentz D.M., Johnson K.A., Karlawish J., Donohue M., Salmon D.P., Aisen P. (2014). The A4 study: Stopping AD before symptoms begin?. Sci. Transl. Med..

[64] Price J.C., Klunk W.E., Lopresti B.J., Lu X., Hoge J.A., Ziolko S.K., Holt D.P., Meltzer C.C., DeKosky S.T., Mathis C.A. (2005). Kinetic modeling of amyloid binding in humans using PET imaging and Pittsburgh Compound-B. J. Cereb. Blood Flow Metab..

[65] Ikonomovic M.D., Klunk W.E., Abrahamson E.E., Mathis C.A., Price J.C., Tsopelas N.D., Lopresti B.J., Ziolko S., Bi W., Paljug W.R. (2008). Post-mortem correlates of *in vivo* PiB-PET amyloid imaging in a typical case of Alzheimer’s disease. Brain.

[66] Lyketsos C.G., Lopez O., Jones B., Fitzpatrick A.L., Breitner J., DeKosky S. (2002). Prevalence of neuropsychiatric symptoms in dementia and mild cognitive impairment: Results from the cardiovascular health study. JAMA.

[67] Seitz D., Purandare N., Conn D. (2010). Prevalence of psychiatric disorders among older adults in long-term care homes: A systematic review. Int. Psychogeriatr..

[68] Cummings J.L., Mega M., Gray K., Rosenberg-Thompson S., Carusi D.A., Gornbein J. (1994). The neuropsychiatric inventory: Comprehensive assessment of psychopathology in dementia. Neurology.

[69] Geda Y.E., Schneider L.S., Gitlin L.N., Miller D.S., Smith G.S., Bell J., Evans J., Lee M., Porsteinsson A., Lanctot K.L. (2013). Neuropsychiatric symptoms in Alzheimer’s disease: Past progress and anticipation of the future. Alzheimer’s Dement..

[70] Ballard C., Howard R. (2006). Neuroleptic drugs in dementia: Benefits and harm. Nat. Rev. Neurosci..

[71] Fischer C.E., Ting W.K., Millikin C.P., Ismail Z., Schweizer T.A., Alzheimer’s Disease Neuroimaging Initiative (2015). Gray matter atrophy in patients with mild cognitive impairment/Alzheimer’s disease over the course of developing delusions. Int. J. Geriatr. Psychiatry.

[72] Ting W.K., Fischer C.E., Millikin C.P., Ismail Z., Chow T.W., Schweizer T.A. (2015). Grey matter atrophy in mild cognitive impairment/early Alzheimer’s disease associated with delusions: A voxel-based morphometry study. Curr. Alzheimer’s Res..

[73] Rafii M.S., Taylor C.S., Kim H.T., Desikan R.S., Fleisher A.S., Katibian D., Brewer J.B., Dale A.M., Aisen P.S. (2014). Neuropsychiatric symptoms and regional neocortical atrophy in mild cognitive impairment and Alzheimer’s disease. Am. J. Alzheimer’s Dis. Dement..

[74] Bruen P.D., McGeown W.J., Shanks M.F., Venneri A. (2008). Neuroanatomical correlates of neuropsychiatric symptoms in Alzheimer’s disease. Brain.

[75] Ismail Z., Nguyen M.Q., Fischer C.E., Schweizer T.A., Mulsant B.H. (2012). Neuroimaging of delusions in Alzheimer’s disease. Psychiatry Res..

[76] Sultzer D.L., Leskin L.P., Melrose R.J., Harwood D.G., Narvaez T.A., Ando T.K., Mandelkern M.A. (2014). Neurobiology of delusions, memory, and insight in Alzheimer’s disease. Am. J. Geriatr. Psychiatry.

[77] Blanc F., Noblet V., Philippi N., Cretin B., Foucher J., Armspach J.P., Rousseau F., Alzheimer’s Disease Neuroimaging Initiative (2014). Right anterior insula: Core region of hallucinations in cognitive neurodegenerative diseases. PLoS ONE.

[78] Donovan N.J., Wadsworth L.P., Lorius N., Locascio J.J., Rentz D.M., Johnson K.A., Sperling R.A., Marshall G.A., Alzheimer’s Disease Neuroimaging Initiative (2014). Regional cortical thinning predicts worsening apathy and hallucinations across the Alzheimer’s disease spectrum. Am. J. Geriatr. Psychiatry.

[79] Cummings J., Mintzer J., Brodaty H., Sano M., Banerjee S., Devanand D.P., Gauthier S., Howard R., Lanctot K., Lyketsos C.G. (2015). Agitation in cognitive disorders: International psychogeriatric association provisional consensus clinical and research definition. Int. Psychogeriatr..

[80] Cohen-Mansfield J., Billig N. (1986). Agitated behaviors in the elderly. I. A conceptual review. J. Am. Geriatr. Soc..

[81] Trzepacz P.T., Yu P., Bhamidipati P.K., Willis B., Forrester T., Tabas L., Schwarz A.J., Saykin A.J., Alzheimer’s Disease Neuroimaging Initiative (2013). Frontolimbic atrophy is associated with agitation and aggression in mild cognitive impairment and Alzheimer’s disease. Alzheimer’s Dement..

[82] Balthazar M.L., Pereira F.R., Lopes T.M., da Silva E.L., Coan A.C., Campos B.M., Duncan N.W., Stella F., Northoff G., Damasceno B.P. (2014). Neuropsychiatric symptoms in Alzheimer’s disease are related to functional connectivity alterations in the salience network. Hum. Brain Mapp..

[83] Rosenberg P.B., Nowrangi M.A., Lyketsos C.G. (2015). Neuropsychiatric symptoms in Alzheimer’s disease: What might be associated brain circuits?. Mol. Asp. Med..

[84] Marin R.S., Wilkosz P.A. (2005). Disorders of diminished motivation. J. Head Trauma Rehabil..

[85] Starkstein S.E., Mayberg H.S., Preziosi T.J., Andrezejewski P., Leiguarda R., Robinson R.G. (1992). Reliability, validity, and clinical correlates of apathy in parkinson’s disease. J. Neuropsychiatry Clin. Neurosci..

[86] Delrieu J., Desmidt T., Camus V., Sourdet S., Boutoleau-Bretonniere C., Mullin E., Vellas B., Payoux P., Lebouvier T., Alzheimer’s Disease Neuroimaging Initiative (2015). Apathy as a feature of prodromal Alzheimer’s disease: An FDG-PET ADNI study. Int. J. Geriatr. Psychiatry.

[87] Lanctot K.L., Moosa S., Herrmann N., Leibovitch F.S., Rothenburg L., Cotter A., Black S.E. (2007). A spect study of apathy in Alzheimer’s disease. Dement. Geriatr. Cogn. Disord..

[88] Murray P.S., Kirkwood C.M., Gray M.C., Fish K.N., Ikonomovic M.D., Hamilton R.L., Kofler J.K., Klunk W.E., Lopez O.L., Sweet R.A. (2014). Hyperphosphorylated tau is elevated in Alzheimer’s disease with psychosis. J. Alzheimer’s Dis..

[89] Koppel J., Sunday S., Goldberg T.E., Davies P., Christen E., Greenwald B.S., Alzheimer’s Disease Neuroimaging Initiative (2014). Psychosis in Alzheimer’s disease is associated with frontal metabolic impairment and accelerated decline in working memory: Findings from the Alzheimer’s disease neuroimaging initiative. Am. J. Geriatr. Psychiatry.

[90] Neary D., Snowden J.S., Gustafson L., Passant U., Stuss D., Black S., Freedman M., Kertesz A., Robert P.H., Albert M. (1998). Frontotemporal lobar degeneration: A consensus on clinical diagnostic criteria. Neurology.

[91] Rascovsky K., Hodges J.R., Kipps C.M., Johnson J.K., Seeley W.W., Mendez M.F., Knopman D., Kertesz A., Mesulam M., Salmon D.P. (2007). Diagnostic criteria for the behavioral variant of frontotemporal dementia (bvFTD): Current limitations and future directions. Alzheimer’s Dis. Assoc. Disord..

[92] Seeley W.W. (2008). Selective functional, regional, and neuronal vulnerability in frontotemporal dementia. Curr. Opin. Neurol..

[93] McKeith I.G., Dickson D.W., Lowe J., Emre M., O’Brien J.T., Feldman H., Cummings J., Duda J.E., Lippa C., Perry E.K. (2005). Diagnosis and management of dementia with lewy bodies: Third report of the dlb consortium. Neurology.

[94] Leucht S., Tardy M., Komossa K., Heres S., Kissling W., Salanti G., Davis J.M. (2012). Antipsychotic drugs *versus* placebo for relapse prevention in schizophrenia: A systematic review and meta-analysis. Lancet.

[95] Palaniyappan L., Liddle P.F. (2012). Does the salience network play a cardinal role in psychosis? An emerging hypothesis of insular dysfunction. J. Psychiatry Neurosci..

[96] Manoliu A., Riedl V., Zherdin A., Mühlau M., Schwerthöffer D., Scherr M., Peters H., Zimmer C., Förstl H., Bäuml J. (2013). Aberrant dependence of default mode/central executive network interactions on anterior insular salience network activity in schizophrenia. Schizophr. Bull..

[97] Moran L.V., Tagamets M.A., Sampath H., O’Donnell A., Stein E.A., Kochunov P., Hong L.E. (2013). Disruption of anterior insula modulation of large-scale brain networks in schizophrenia. Biol. Psychiatry.

[98] Palaniyappan L., Simmonite M., White T.P., Liddle E.B., Liddle P.F. (2013). Neural primacy of the salience processing system in schizophrenia. Neuron.

[99] Wotruba D., Michels L., Buechler R., Metzler S., Theodoridou A., Gerstenberg M., Walitza S., Kollias S., Rössler W., Heekeren K. (2014). Aberrant coupling within and across the default mode, task-positive, and salience network in subjects at risk for psychosis. Schizophr. Bull..

[100] Driesen N.R., McCarthy G., Bhagwagar Z., Bloch M., Calhoun V., D’Souza D.C., Gueorguieva R., He G., Ramachandran R., Suckow R.F. (2013). Relationship of resting brain hyperconnectivity and schizophrenia-like symptoms produced by the NMDA receptor antagonist ketamine in humans. Mol. Psychiatry.

[101] Fuchigami T., Nakayama M., Yoshida S. (2015). Development of PET and SPECT probes for glutamate receptors. Sci. World J..

[102] Shen L.H., Liao M.H., Tseng Y.C. (2012). Recent advances in imaging of dopaminergic neurons for evaluation of neuropsychiatric disorders. J. Biomed. Biotechnol..

[103] Poels E.M., Kegeles L.S., Kantrowitz J.T., Slifstein M., Javitt D.C., Lieberman J.A., Abi-Dargham A., Girgis R.R. (2014). Imaging glutamate in schizophrenia: Review of findings and implications for drug discovery. Mol. Psychiatry.

[104] Agius M., Goh C., Ulhaq S., McGorry P. (2010). The staging model in schizophrenia, and its clinical implications. Psychiatr. Danub..

[105] Marsman A., van den Heuvel M.P., Klomp D.W.J., Kahn R.S., Luijten P.R., Hulshoff Pol H.E. (2013). Glutamate in schizophrenia: A focused review and meta-analysis of ^1^H-MRS studies. Schizophr. Bull..

[106] Fryer S.L., Woods S.W., Kiehl K.A., Calhoun V.D., Pearlson G.D., Roach B.J., Ford J.M., Srihari V.H., McGlashan T.H., Mathalon D.H. (2013). Deficient suppression of default mode regions during working memory in individuals with early psychosis and at clinical high-risk for psychosis. Front. Psychiatry.

[107] Zhang F., Qiu L., Yuan L., Ma H., Ye R., Yu F., Hu P., Dong Y., Wang K. (2014). Evidence for progressive brain abnormalities in early schizophrenia: A cross-sectional structural and functional connectivity study. Schizophr. Res..

[108] Benes F.M., Berretta S. (2001). Gabaergic interneurons: Implications for understanding schizophrenia and bipolar disorder. Neuropsychopharmacology.

[109] Lewis D.A., Curley A.A., Glausier J.R., Volk D.W. (2012). Cortical parvalbumin interneurons and cognitive dysfunction in schizophrenia. Trends Neurosci..

[110] Goto N., Yoshimura R., Moriya J., Kakeda S., Ueda N., Ikenouchi-Sugita A., Umene-Nakano W., Hayashi K., Oonari N., Korogi Y. (2009). Reduction of brain gamma-aminobutyric acid (GABA) concentrations in early-stage schizophrenia patients: 3T Proton MRS study. Schizophr. Res..

[111] Taylor S.F., Tso I.F. (2014). GABA abnormalities in schizophrenia: A methodological review of *in vivo* studies. Schizophr. Res..

[112] Anticevic A., Cole M.W., Repovs G., Savic A., Driesen N.R., Yang G., Cho Y.T., Murray J.D., Glahn D.C., Wang X.-J. (2013). Connectivity, pharmacology, and computation: Toward a mechanistic understanding of neural system dysfunction in schizophrenia. Front. Psychiatry.

[113] Wiebking C., Duncan N.W., Qin P., Hayes D.J., Lyttelton O., Gravel P., Verhaeghe J., Kostikov A.P., Schirrmacher R., Reader A.J. (2014). External awareness and GABA—A multimodal imaging study combining fMRI and [18F]flumazenil-PET. Hum. Brain Mapp..

[114] Monji A., Kato T.A., Mizoguchi Y., Horikawa H., Seki Y., Kasai M., Yamauchi Y., Yamada S., Kanba S. (2013). Neuroinflammation in schizophrenia especially focused on the role of microglia. Prog. Neuropsychopharmacol. Biol. Psychiatry.

[115] Lennox B.R., Coles A.J., Vincent A. (2012). Antibody-mediated encephalitis: A treatable cause of schizophrenia. Br. J. Psychiatry.

[116] Sommer I.E., de Witte L., Begemann M., Kahn R.S. (2012). Nonsteroidal anti-inflammatory drugs in schizophrenia: Ready for practice or a good start? A meta-analysis. J. Clin. Psychiatry.

[117] Ory D., Celen S., Verbruggen A., Bormans G. (2014). PET radioligands for *in vivo* visualization of neuroinflammation. Curr. Pharm. Des..

[118] Hasler G., Drevets W.C., Manji H.K., Charney D.S. (2004). Discovering endophenotypes for major depression. Neuropsychopharmacology.

[119] Ressler K.J., Mayberg H.S. (2007). Targeting abnormal neural circuits in mood and anxiety disorders: From the laboratory to the clinic. Nat. Neurosci..

[120] Sobczak S., Honig A., van Duinen M.A., Riedel W.J. (2002). Serotonergic dysregulation in bipolar disorders: A literature review of serotonergic challenge studies. Bipolar Disord..

[121] Heiss W.D., Herholz K. (2006). Brain receptor imaging. J. Nucl. Med..

[122] Ruhe H.G., Mason N.S., Schene A.H. (2007). Mood is indirectly related to serotonin, norepinephrine and dopamine levels in humans: A meta-analysis of monoamine depletion studies. Mol. Psychiatry.

[123] Kessler R.C., Berglund P., Demler O., Jin R., Merikangas K.R., Walters E.E. (2005). Lifetime prevalence and age-of-onset distributions of DSM-IV disorders in the national comorbidity survey replication. Arch. Gen. Psychiatry.

[124] Gershuny B.S., Baer L., Parker H., Gentes E.L., Infield A.L., Jenike M.A. (2008). Trauma and posttraumatic stress disorder in treatment-resistant obsessive-compulsive disorder. Depression Anxiety.

[125] Friedman M.J., Resick P.A., Bryant R.A., Strain J., Horowitz M., Spiegel D. (2011). Classification of trauma and stressor-related disorders in DSM-5. Depression Anxiety.

[126] Peterson A., Thome J., Frewen P., Lanius R.A. (2014). Resting-state neuroimaging studies: A new way of identifying differences and similarities among the anxiety disorders?. Can. J. Psychiatry..

[127] Rabinak C.A., Angstadt M., Welsh R.C., Kenndy A.E., Lyubkin M., Martis B., Phan K.L. (2011). Altered amygdala resting-state functional connectivity in post-traumatic stress disorder. Front. Psychiatry.

[128] Sripada R.K., King A.P., Welsh R.C., Garfinkel S.N., Wang X., Sripada C.S., Liberzon I. (2012). Neural dysregulation in posttraumatic stress disorder: Evidence for disrupted equilibrium between salience and default mode brain networks. Psychosom. Med..

[129] Qiu C., Liao W., Ding J., Feng Y., Zhu C., Nie X., Zhang W., Chen H., Gong Q. (2011). Regional homogeneity changes in social anxiety disorder: A resting-state fMRI study. Psychiatry Res..

[130] Hahn A., Stein P., Windischberger C., Weissenbacher A., Spindelegger C., Moser E., Kasper S., Lanzenberger R. (2011). Reduced resting-state functional connectivity between amygdala and orbitofrontal cortex in social anxiety disorder. NeuroImage.

[131] Liao W., Qiu C., Gentili C., Walter M., Pan Z., Ding J., Zhang W., Gong Q., Chen H. (2010). Altered effective connectivity network of the amygdala in social anxiety disorder: A resting-state fMRI study. PLoS ONE.

[132] Peng Z., Shi F., Shi C., Yang Q., Chan R.C., Shen D. (2014). Disrupted cortical network as a vulnerability marker for obsessive-compulsive disorder. Brain Struct. Funct..

[133] Stern E.R., Fitzgerald K.D., Welsh R.C., Abelson J.L., Taylor S.F. (2012). Resting-state functional connectivity between fronto-parietal and default mode networks in obsessive-compulsive disorder. PLoS ONE.

[134] Fitzgerald K.D., Stern E.R., Angstadt M., Nicholson-Muth K.C., Maynor M.R., Welsh R.C., Hanna G.L., Taylor S.F. (2010). Altered function and connectivity of the medial frontal cortex in pediatric obsessive-compulsive disorder. Biol. Psychiatry.

[135] Gusnard D.A., Akbudak E., Shulman G.L., Raichle M.E. (2001). Medial prefrontal cortex and self-referential mental activity: Relation to a default mode of brain function. Proc. Natl. Acad. Sci. USA.

[136] Li P., Li S., Dong Z., Luo J., Han H., Xiong H., Guo Z., Li Z. (2012). Altered resting state functional connectivity patterns of the anterior prefrontal cortex in obsessive-compulsive disorder. NeuroReport.

[137] Posner J., Hellerstein D.J., Gat I., Mechling A., Klahr K., Wang Z., McGrath P.J., Stewart J.W., Peterson B.S. (2013). Antidepressants normalize the default mode network in patients with dysthymia. JAMA Psychiatry.

[138] Muller J., Roberts J.E. (2005). Memory and attention in obsessive-compulsive disorder: A review. J. Anxiety Disord..

[139] Hamner M.B., Robert S., Frueh B.C. (2004). Treatment-resistant posttraumatic stress disorder: Strategies for intervention. CNS Spectr..

[140] Ammar G., Naja W.J., Pelissolo A. (2015). Treatment-resistant anxiety disorders: A literature review of drug therapy strategies. L’Encephale.

[141] Magalhaes P.V., Kapczinski N.S., Kapczinski F. (2010). Correlates and impact of obsessive-compulsive comorbidity in bipolar disorder. Compr. Psychiatry.

[142] Al-Asadi A.M., Klein B., Meyer D. (2014). Comorbidity structure of psychological disorders in the online e-pass data as predictors of psychosocial adjustment measures: Psychological distress, adequate social support, self-confidence, quality of life, and suicidal ideation. J. Med. Internet Res..

[143] Lopez-Leon S., Janssens A.C., Gonzalez-Zuloeta Ladd A.M., Del-Favero J., Claes S.J., Oostra B.A., van Duijn C.M. (2008). Meta-analyses of genetic studies on major depressive disorder. Mol. Psychiatry.

[144] Mill J., Petronis A. (2007). Molecular studies of major depressive disorder: The epigenetic perspective. Mol. Psychiatry.

[145] Mulders P.C., van Eijndhoven P.F., Schene A.H., Beckmann C.F., Tendolkar I. (2015). Resting-state functional connectivity in major depressive disorder: A review. Neurosci. Biobehav. Rev..

[146] Krishnan V., Nestler E.J. (2008). The molecular neurobiology of depression. Nature.

[147] Lee B.H., Park Y.M., Um T.H., Kim S. (2014). Lower serum brain-derived neurotrophic factor levels are associated with failure to achieve remission in patients with major depression after escitalopram treatment. Neuropsychiatr. Dis. Treat..

[148] Miller C.H., Hamilton J.P., Sacchet M.D., Gotlib I.H. (2015). Meta-analysis of functional neuroimaging of major depressive disorder in youth. JAMA Psychiatry.

[149] Pizzagalli D.A. (2011). Frontocingulate dysfunction in depression: Toward biomarkers of treatment response. Neuropsychopharmacology.

[150] Drevets W.C., Price J.L., Furey M.L. (2008). Brain structural and functional abnormalities in mood disorders: Implications for neurocircuitry models of depression. Brain Struct. Funct..

[151] Kaiser R.H., Andrews-Hanna J.R., Wager T.D., Pizzagalli D.A. (2015). Large-scale network dysfunction in major depressive disorder: A meta-analysis of resting-state functional connectivity. JAMA Psychiatry.

[152] Hasler G., Northoff G. (2011). Discovering imaging endophenotypes for major depression. Mol. Psychiatry.

[153] Chen Y., Wang C., Zhu X., Tan Y., Zhong Y. (2015). Aberrant connectivity within the default mode network in first-episode, treatment-naive major depressive disorder. J. Affect. Disord..

[154] Peng D., Liddle E.B., Iwabuchi S.J., Zhang C., Wu Z., Liu J., Jiang K., Xu L., Liddle P.F., Palaniyappan L. (2015). Dissociated large-scale functional connectivity networks of the precuneus in medication-naive first-episode depression. Psychiatry Res..

[155] Shen Y., Yao J., Jiang X., Zhang L., Xu L., Feng R., Cai L., Liu J., Wang J., Chen W. (2015). Sub-hubs of baseline functional brain networks are related to early improvement following two-week pharmacological therapy for major depressive disorder. Hum. Brain Mapp..

[156] McGrath C.L., Kelley M.E., Holtzheimer P.E., Dunlop B.W., Craighead W.E., Franco A.R., Craddock R.C., Mayberg H.S. (2013). Toward a neuroimaging treatment selection biomarker for major depressive disorder. JAMA Psychiatry.

[157] Woods S.W. (2000). The economic burden of bipolar disease. J. Clin. Psychiatry.

[158] Oswald P., Souery D., Kasper S., Lecrubier Y., Montgomery S., Wyckaert S., Zohar J., Mendlewicz J. (2007). Current issues in bipolar disorder: A critical review. Eur. Neuropsychopharmacol..

[159] Baldassano C.F., Ballas C.A., O’Reardon J.P. (2004). Rethinking the treatment paradigm for bipolar depression: The importance of long-term management. CNS Spectr..

[160] Almeida O.P., Garrido G.J., Etherton-Beer C., Lautenschlager N.T., Arnolda L., Alfonso H., Flicker L. (2013). Brain and mood changes over 2 years in healthy controls and adults with heart failure and ischaemic heart disease. Eur. J. Heart Fail..

[161] Belmaker R.H. (2004). Bipolar disorder. N. Engl. J. Med..

[162] Lachaine J., Beauchemin C., Mathurin K., Gilbert D., Beillat M. (2014). Cost-effectiveness of asenapine in the treatment of bipolar disorder in Canada. BMC Psychiatry.

[163] De Jesus J.R., de Campos B.K., Galazzi R.M., Martinez J.L., Arruda M.A. (2015). Bipolar disorder: Recent advances and future trends in bioanalytical developments for biomarker discovery. Anal. Bioanal. Chem..

[164] Calhoun V.D., Maciejewski P.K., Pearlson G.D., Kiehl K.A. (2008). Temporal lobe and “default” hemodynamic brain modes discriminate between schizophrenia and bipolar disorder. Hum. Brain Mapp..

[165] Magioncalda P., Martino M., Conio B., Escelsior A., Piaggio N., Presta A., Marozzi V., Rocchi G., Anastasio L., Vassallo L. (2015). Functional connectivity and neuronal variability of resting state activity in bipolar disorder—Reduction and decoupling in anterior cortical midline structures. Hum. Brain Mapp..

[166] Murray R.M., Sham P., van Os J., Zanelli J., Cannon M., McDonald C. (2004). A developmental model for similarities and dissimilarities between schizophrenia and bipolar disorder. Schizophr. Res..

[167] Weiser M., Reichenberg A., Rabinowitz J., Kaplan Z., Mark M., Bodner E., Nahon D., Davidson M. (2001). Association between nonpsychotic psychiatric diagnoses in adolescent males and subsequent onset of schizophrenia. Arch. Gen. Psychiatry.

[168] Chai X.J., Whitfield-Gabrieli S., Shinn A.K., Gabrieli J.D., Nieto Castanon A., McCarthy J.M., Cohen B.M., Ongur D. (2011). Abnormal medial prefrontal cortex resting-state connectivity in bipolar disorder and schizophrenia. Neuropsychopharmacology.

[169] Ongur D., Lundy M., Greenhouse I., Shinn A.K., Menon V., Cohen B.M., Renshaw P.F. (2010). Default mode network abnormalities in bipolar disorder and schizophrenia. Psychiatry Res..

[170] Meda S.A., Gill A., Stevens M.C., Lorenzoni R.P., Glahn D.C., Calhoun V.D., Sweeney J.A., Tamminga C.A., Keshavan M.S., Thaker G. (2012). Differences in resting-state functional magnetic resonance imaging functional network connectivity between schizophrenia and psychotic bipolar probands and their unaffected first-degree relatives. Biol. Psychiatry.

[171] Mamah D., Barch D.M., Repovs G. (2013). Resting state functional connectivity of five neural networks in bipolar disorder and schizophrenia. J. Affect. Disord..

[172] Argyelan M., Ikuta T., DeRosse P., Braga R.J., Burdick K.E., John M., Kingsley P.B., Malhotra A.K., Szeszko P.R. (2014). Resting-state fMRI connectivity impairment in schizophrenia and bipolar disorder. Schizophr. Bull..

[173] Schwenzer N.F., Stegger L., Bisdas S., Schraml C., Kolb A., Boss A., Muller M., Reimold M., Ernemann U., Claussen C.D. (2012). Simultaneous PET/MR imaging in a human brain PET/MR system in 50 patients—Current state of image quality. Eur. J. Radiol..

[174] Dyson F.J. (2012). History of science. Is science mostly driven by ideas or by tools?. Science.

[175] Anazodo U.C., Thiessen J.D., Ssali T., Mandel J., Gunther M., Butler J., Pavlosky W., Prato F.S., Thompson R.T., St Lawrence K.S. (2014). Feasibility of simultaneous whole-brain imaging on an integrated PET-MRI system using an enhanced 2-point dixon attenuation correction method. Front. Neurosci..

[176] Mach R.H. (2014). New targets for the development of PET tracers for imaging neurodegeneration in Alzheimer’s disease. J. Nucl. Med..

[177] Shen Q., Jiang M., Luo J. (2011). An improved auto-window algorithm for MR image. Zhongguo Yi Liao Qi Xie Za Zhi.

[178] Vallabhajosula S. (2009). Molecular Imaging. Radiopharmaceuticals for PET and SPECT.

[179] Lohoff F.W. (2010). Overview of the genetics of major depressive disorder. Curr. Psychiatry Rep..

[180] Zhang D., Wang Y., Zhou L., Yuan H., Shen D., Alzheimer’s Disease Neuroimaging Initiative (2011). Multimodal classification of Alzheimer’s disease and mild cognitive impairment. NeuroImage.

[181] Villemagne V.L., Fodero-Tavoletti M.T., Masters C.L., Rowe C.C. (2015). Tau imaging: Early progress and future directions. Lancet Neurol..

[182] Jack C.R., Knopman D.S., Jagust W.J., Petersen R.C., Weiner M.W., Aisen P.S., Shaw L.M., Vemuri P., Wiste H.J., Weigand S.D. (2013). Update on hypothetical model of Alzheimer’s disease biomarkers. Lancet Neurol..

[183] Shah Y.B., Marsden C.A. (2004). The application of functional magnetic resonance imaging to neuropharmacology. Curr. Opin. Pharmacol..

[184] Andreasen N.C., Nopoulos P., Magnotta V., Pierson R., Ziebell S., Ho B.-C. (2011). Progressive brain change in schizophrenia: A prospective longitudinal study of first-episode schizophrenia. Biol. Psychiatry.

[185] Parsey R.V. (2010). Serotonin receptor imaging: Clinically useful?. J. Nucl. Med..

[186] Hiemke C. (2008). Therapeutic drug monitoring in neuropsychopharmacology: Does it hold its promises?. Eur. Arch. Psychiatry Clin. Neurosci..

[187] Dazzan P., Morgan K.D., Orr K., Hutchinson G., Chitnis X., Suckling J., Fearon P., McGuire P.K., Mallett R.M., Jones P.B. (2005). Different effects of typical and atypical antipsychotics on grey matter in first episode psychosis: The aesop study. Neuropsychopharmacology.

[188] Crow T.J. (2008). The emperors of the schizophrenia polygene have no clothes. Psychol. Med..

[189] Bassett A.S., Scherer S.W., Brzustowicz L.M. (2010). Copy number variations in schizophrenia: Critical review and new perspectives on concepts of genetics and disease. Am. J. Psychiatry.

[190] Kambeitz J.P., Howes O.D. (2015). The serotonin transporter in depression: Meta-analysis of *in vivo* and post mortem findings and implications for understanding and treating depression. J. Affect. Disord..

